# HIRA Is Required for Heart Development and Directly Regulates *Tnni2* and *Tnnt3*

**DOI:** 10.1371/journal.pone.0161096

**Published:** 2016-08-12

**Authors:** Daniel Dilg, Rasha Noureldin M. Saleh, Sarah Elizabeth Lee Phelps, Yoann Rose, Laurent Dupays, Cian Murphy, Timothy Mohun, Robert H. Anderson, Peter J. Scambler, Ariane L. A. Chapgier

**Affiliations:** 1 Developmental Biology of Birth Defects Section, Institute of Child Health, University College London, 30 Guilford Street, London, WC1N 1EH, United Kingdom; 2 Faculty of Medicine, Alexandria University, El-Gaish Rd, Alexandria, Egypt; 3 The Francis Crick Institute, Mill Hill Laboratory, the Ridgeway, Mill Hill, London NW7 1AA, United Kingdom; 4 UCL Genetics Institute (UGI) Department of Genetics, Environment and Evolution University College London, Gower St, London WC1E 6BT, United Kingdom; 5 Institute of Genetic Medicine, Newcastle University, International Centre for Life, Central Parkway, Newcastle upon Tyne, NE1 3BZ, United Kingdom; Rutgers University Newark, UNITED STATES

## Abstract

Chromatin remodelling is essential for cardiac development. Interestingly, the role of histone chaperones has not been investigated in this regard. HIRA is a member of the HUCA (HIRA/UBN1/CABIN1/ASF1a) complex that deposits the variant histone H3.3 on chromatin independently of replication. Lack of HIRA has general effects on chromatin and gene expression dynamics in embryonic stem cells and mouse oocytes. Here we describe the conditional ablation of *Hira* in the cardiogenic mesoderm of mice. We observed surface oedema, ventricular and atrial septal defects and embryonic lethality. We identified dysregulation of a subset of cardiac genes, notably upregulation of troponins *Tnni2* and *Tnnt3*, involved in cardiac contractility and decreased expression of *Epha3*, a gene necessary for the fusion of the muscular ventricular septum and the atrioventricular cushions. We found that HIRA binds GAGA rich DNA loci in the embryonic heart, and in particular a previously described enhancer of *Tnni2/Tnnt3* (*TTe*) bound by the transcription factor NKX2.5. HIRA-dependent H3.3 enrichment was observed at the *TTe* in embryonic stem cells (ESC) differentiated toward cardiomyocytes *in vitro*. Thus, we show here that HIRA has locus-specific effects on gene expression and that histone chaperone activity is vital for normal heart development, impinging on pathways regulated by an established cardiac transcription factor.

## Introduction

The heart is the first organ to be formed that is vital for embryogenesis. In the post-gastrulation embryo at embryonic day (E) 6.5, the mesodermally derived cardiac crescent appears and undergoes a series of regulated morphological changes leading to a linear heart tube [1]. After heart looping at E9.5, the four chambers are progressively septated. By E15.5 the heart is fully functional. Cardiovascular development is tightly regulated by dynamic gene expression. Epigenetic modifications such as post-translational histone modification have been described to influence development and differentiation [2]. However, little is known of the role of histone chaperone functions during cardiac development.

HIRA is a component of the HUCA complex that deposits the variant histone H3.3 into chromatin independently of cellular replication [3], influencing transcription [3–5], genome integrity [6], fertilization [7], cellular senescence [8], and genome reprogramming [9]. In mouse embryonic stem cells (ESCs), HIRA deposits H3.3 predominantly in genic regions, but also at a subset of enhancer and intergenic regions [4]. *Hira* null embryos display a range of developmental defects during and subsequent to gastrulation [10]. A small proportion of these mutants survived to E10.5 and showed abnormal heart looping and substantial pericardial oedema amongst other defects including abnormal placentation suggesting that the heart defects may have been a secondary effect.

In order to assess the role of HIRA in cardiovascular development, we used a conditional allele of *Hira* in mice in conjunction with various relevant cardiac relevant CRE recombinases to bypass the early lethality of *Hira* null embryos. *Mesp1* is the earliest known marker of cardiac progenitors which give rise to cardiomyocytes, endothelial cells (ECs), epicardial derived cells and smooth muscle cells. We employed *Mesp1Cre* to target *Hira* in these early cardiac progenitors, and then used *Nkx2*.*5Cre*, *Mef2cCre* and *Tie2Cre* drivers to refine requirements of HIRA in the second heart field (SHF) and endothelial lineages.

We show here that HIRA plays a major role in the cardiogenic mesoderm. *Mesp1* conditionally mutant *Hira* embryos presented with generalised oedema and cardiac malformations such as ventricular septal defect (VSD), atrial septal defect (ASD), thin ventricular wall and constricted pulmonary trunk (PT). Using RNAseq we report that, of the most significantly changed genes, absence of HIRA impacts troponins known to be relevant for regulation of muscle contractility, and *Epha3* required for the endothelial to mesenchymal transition (EMT) taking place in the atrioventricular cushions prior to septation and valve formation. Quantitative Chromatin Immunoprecipitation (qChIP) and ChIP followed by sequencing (ChIPseq) analyses show that HIRA is strongly enriched at the common enhancer of troponins *Tnni2* and *Tnnt3* (the *TTe* site) in E12.5 Wild Type (WT) hearts. ESCs differentiated towards cardiomyocytes confirmed this specific HIRA enrichment at the *TTe* site, associated with HIRA-dependent H3.3 deposition. The *TTe* site has been shown to be bound by NKX2.5, as determined by previous ChIP in embryonic hearts [11], and DamID experiments using HL-1 cells [12]. In summary we provide the first indication that histone chaperone complexes have a role in cardiovascular development and suggest that HIRA complexes directly regulate a subset of genes vital for cardiovascular morphogenesis.

## Methods

### Mouse lines

Animal maintenance, husbandry and procedures were carried out in accordance with British Home Office regulations. *Hira* knockout mice have been described previously [10]. The *Hira* pre-conditional allele was generated by the Wellcome Trust Sanger Institute: *Hiratm1a*(EUCOMM) Wtsi, MGI:4431679. The Cre line used were: *Mesp1Cre* (MGI:2176467; *Mesp1tm2*(cre)Ysa), *Mef2CCre* (MGI:3639735 Tg(*Mef2c-cre*)2Blk), *Wnt1Cre* (MGI:2386570; Tg(*Wnt1-cre*)11Rth), *Nkx2*.*5Cre* (MGI:2654594; *Nkx2-5tm1*(cre)Rjs), *Tie2Cre* (MGI: 2450311,Tg(*Tek-cre*)1Ywa). All lines were maintained on a CD1-ICR background.

### Optical projection tomography

Embryos were fixed overnight in 4% PFA/PBS and mounted in low-melting agarose (Life Technologies). Up to E14.5, whole embryos were processed. At E15.5, the trunks were opened and cartilage from the rib cage was discarded to help with the subsequent scanning since cartilage does not clear completely. Samples were then trimmed to remove the excess of agarose and washed in 100% methanol followed by clearing in benzyl alcohol:benzyl benzoate (BABB). Scanning was undertaken using a Bioptonics OPT Scanner 3001M (MRC Technology, Edinburgh, UK). NRecon software (Skyscan NV) was used for image reconstruction from projections using a back-projection algorithm. FIJI (Image J) and Volocity were used for image analysis and 3D reconstruction.

### RNA extraction and sequencing

RNA extraction was done in triplicate from *Mesp1Cre*;*Hira*^*-/fl*^ and *Mesp1Cre*;*Hira*^*+/fl*^ embryonic hearts at E11.5 and E12.5 using the QIAGEN RNeasy mini kit (74104). RNA QC was performed by a 2100 bioanalyzer. RNAseq was processed by Illumina NextSeq 500, and paired ends reads were produced. Reads were aligned and normalised using BOWTIE and DEseq R package. Strand NGS 2.5 software, which uses the DEseq algorithm, was used to incorporate additional downstream analysis such as Gene Ontology. The Mann Whitney unpaired test and Benjamini Hochberg False discovery rate (FDR) were applied. The genes were sorted using the following settings: adjusted p-value ≤ 0.05 and absolute fold change ≥ 1.5. We found 95% of similar results between the two analysis methods.

### Reverse transcription and quantitative real time PCR

The High-Capacity RNA-to-cDNA^™^ Kit (Thermo fisher 4387406) was used to obtain cDNA from the RNA (see above) for the qRT-PCR experiments, according to the manufacturer’s instructions. Primers for qRT-PCR were designed using primer-blast (http://www.ncbi.nlm.nih.gov/tools/primer-blast/) with the following option: primers must span an exon-exon junction and be separated by at least one intron, thus ensuring amplification of cDNA and not possible gDNA contamination. The PCR product size was set to be between 80 and 160 bp. The standard curve method was performed using SYBR green and results normalised to *Gapdh*. The CFX96 Touch^™^ Real-Time PCR Detection System was used. Following the reaction, melting curves were checked and samples were run on an agarose gel to verify the amplimer size.

### *In situ* hybridisation on paraffin sections

The following plasmids were used: *Tnni2*, Image clone 1448494, *Epha3* Pblu2KSP. They were kindly provided by Tim Mohun and Jeffrey Bush respectively. Briefly, plasmids were linearized using EcoRI and XhoI respectively and RNA synthesised using T3 and T7 RNA polymerase respectively. RNA was extracted from a 1% agarose gel using the QIAquick Gel Extraction Kit (Qiagen). 1 μg of linearised plasmid was used for *in vitro* transcription of probes using a DIG RNA labelling kit (Roche). Probes were purified by precipitation with the addition of 2 μl 0.5M EDTA (pH 8), 5 μl 4 M LiCl and 150 μl ethanol to the reaction and centrifugation of the precipitates.

Paraffin sections were prepared as follow. Briefly, slides were incubated in 20 μg/ml Proteinase K (Sigma-Aldrich) for 8 minutes, washed in 2 mg/ml glycine then PBS, then fixed in 4% PFA/PBS for 20 minutes. Following further PBS washes they were incubated for 1 hour at 70°C in a humidified chamber in hybridisation buffer (50% formamide, 5X SSC pH 4.5, 50 μg/ml yeast RNA, 1% SDS, 50 μg/ml heparin) followed by overnight incubation in hybridisation buffer containing between 1 and 2 μg/ml of antisense RNA probe. Slides were then rinsed twice in 2X SSC buffer pH4.5, followed by three washes at 65°C in Solution I (50% formamide, 5X SSC pH4.5, 1% SDS), two washes in Solution II (50% formamide, 2X SSC pH4.5) and finally two washes at RT in MABT (0.1 M maleic acid, 0.15 M NaCl, 0.01% Tween-20, 2 mM Levamisole (Sigma-Aldrich), pH7.5). Slides were then incubated in blocking solution (2% Boehringer Blocking Reagent (Roche), 10% sheep serum in MABT) for 1 hour followed by overnight incubation at 4°C with an alkaline-phosphatase (AP) conjugated anti-DIG antibody (Roche) diluted 1:2000 in blocking buffer. Following further washes in MABT and AP buffer (100 mM Tris, pH 9.5, 50 mM MgCl2, 100 mM NaCl, 0.1% Tween-20, 2 mM Levamisol), AP activity was detected using BM Purple (Roche) for at least 24 hours.

### HIRA qChIP

30 to 40 E12.5 WT hearts were pooled, washed in PBS and cross-linked for 45 min with 1.5 mM of EGS (Sigma, E3257), followed by 15 min of 1% formaldehyde (from a freshly made filtered stock at 18.5%) at 37°C. The reaction was quenched by the addition of 125 mM of Glycine, left for 15 min at RT. Hearts were lysed in 50 mM Hepes-KOH pH 7.5, 140 mM NaCl, 1 mM EDTA, 10% glycerol, 0.5% NP-40, 0.25% Triton, 1X anti-protease cocktail (Roche, 04693132001), 1 mM PMSF for 10 min at 4°C (LB1). The hearts were briefly spun down and resuspended in 10 mM Tris-HCL pH 8.0, 200 mM NaCl, 1 mM EDTA, 0.5 mM EGTA, 1X anti-protease cocktail, 1 mM PMSF for 10 min at 4°C (LB2). Finally the hearts were resuspended in10mM Tris-HCL pH 8.0, 100mM NaCl, 1mM EDTA, 0.5mM EGTA, 0.1% DOC, 0.5% N-Lauroylsarcosine, 1X anti-protease cocktail and 1mM PMSF (LB3) rolling O/N at 4°C. 5 min sonication at 5 μA with Soniprep 150 MS was completed 30 times, with 5 min on ice in between. Antibodies were coupled to magnetic beads (Dynabeads, InVitrogen, 112.03D) for at least 4 hours at 4C and washed 3 times in LB3. 10% of input was isolated and protein–DNA complexes were immunoprecipitated using WC15 antibody against HIRA, rolling overnight. Beads were then washed once with 20 mM tris pH 8.0, 150 mM NaCl, 0.1% SDS, 1% Triton, 2 mM EDTA (WB1), once with 20 mM Tris pH 8.0, 500 mM NaCl, 0.1% SDS, 1% Triton, 2mM EDTA (WB2), once with 10 mM Tris pH 8.0, 150 mM LiCl, 1% NP-40, 1% DOC, 1 mM EDTA, then TE 10:1, 50 mM NaCl (WB3), and finally in TE 10:1. Samples were treated overnight with 50 mM Tris pH 8.0, 10 mM EDTA and 1% SDS at 65°C (EB), then with RNAse (Qiagen, 19101) for an hour at 25°C and with PK for 2h at 56°C. DNA was purified with QIAGEN’s PCR purification kit (28104). Purified DNA was quantified by quantitative PCR, using the purified input chromatin as a positive control. ChIP enrichment was calculated by normalisation to the Input signal (= 100%).

### NKX2.5 qChIP

E12.5 embryos were dissected in PBS. Hearts were flash frozen and stored at -80°C during genotyping. 20 WT hearts and 20 *Mesp1CreHira*^*fl/*^ hearts were pooled respectively and fixed for 15 min in 1% formaldehyde at 37°C then quenched by 125 mM of Glycine for 15 min at RT. Hearts were lysed in RIPA buffer (50 mM Tris pH7, 150 mM NaCl, 1% NP-40, 1 mM EDTA, 50 mM NaF, 0.5% DOC, 0.1% SD. 2 μM sodium orthovanadate, protease inhibitor cocktail 1X (Roche) and PMSF 1 mM were added prior to the experiment. The hearts in lysis buffer were placed on a rotating wheel at 4°C overnight. A syringe (25G) was used to finish the lysis. The lysates were then sonicated (10 rounds of 1 min of sonication at 5 μA with Soniprep 150 MS, with 1 min on ice in between). The protein G beads were incubated overnight at 4°C in PBS with 10 μg of NKX2.5 antibody (N-19 Santa Cruz) and washed 3x in the previous RIPA buffer. They were then incubated with the sonicated chromatin at 4°C O/N. The following day, the beads were washed 2X for 5min in WB1 (10 mM HEPES pH 7.6, 1 mM EDTA, 0.5 mM EGTA, 0.25% Triton X-100) and 2X for 5 min in WB2 (10 mM HEPES pH 7.6, 200 mM NaCl, 1 mM EDTA, 0.5 mM EGTA, 0.01% Triton X-100). The samples were then processed the same way as for HIRA qChIP.

### Library preparation, Sequencing and Analysis for HIRA ChIPseq

Libraries were prepared using the NEB DNA Ultra kit, with a selection of fragments size of ~200bp. They were sequenced on the Illumina NextSeq 500, v2 chemistry and produced paired ends. Alignment was done using bowtie2 with mm10. Peak detection and consensus sequence discovery was undertaken using Strand NGS software 2.5, which includes the algorithm of MACS1.4 (p≤10^−4^, other settings left as default) (Model-based Analysis for ChIPseq), after removal of poor quality and duplicate sequences normalisation was done using RPKM. Lists of genes within +/- 5Kb of the TSS and TES was generated using Strand NGS. Bedtools intersect tool was used to define the overlapping peaks between different BED files. In addition, PAPST (Peak Assignment and Profile Search Tool) software was used to overlap regions of interest and establish genome wide enrichment patterns of HIRA ChIPseq [13].

### Embryonic stem cell culture and differentiation to cardiomyocytes

H3.3-HA tagged wild type (W9.5) and *Hira*-null (Clone 104) mESCs have been previously described [5]. They were maintained in an undifferentiated state on 0.1% gelatin coated flasks in Knockout^™^ D-MEM (GIBCO, 10829), supplemented with 15% ES-FCS (Millipore ES-009B), 1X Glutamax (GIBCO 35050–038), 1X Penicillin/Streptomycin (GIBCO 15140), 1X MEM NEAA (GIBCO 11140–035), 0.1 mM 2-β-mercaptoethanol (SIGMA M-7522) and 103 Units/ml LIF (Millipore, ESG-1106) at 37°C and 5% CO2. These cells were differentiated using the well-described hanging-drop method [14] at a concentration of 25 cells/μl in DMEM (GIBCO 61965–026), complemented with 15% ES-FCS (Millipore ES-009B), 1x Penicillin/Streptomycin (GIBCO 15140), 1x MEM NEAA (GIBCO 11140–035), 0.1mM 2-mercaptoethanol (SIGMA M-7522). Cells were detached and plated on regular gelatin coated TC plates at day 4 of differentiation.

### Immunofluorescence

Paraffin sections were rinsed in PBS and permeabilised with 0.5% Triton X-100 for 10 mins at RT. Then rinsed twice in PBS. Blocking was accomplished with 1% BSA, 10% sheep serum and 0.1% Triton X-100 for 1 hour at RT. Slides were incubated with primary antibody (1:100 of Troponin C: ab30807) diluted in block buffer, then washed 3X in PBS + 0.1% Triton X-100 followed by 2 rinses in PBS. Incubation with secondary antibody was done diluted in block (1:200) for 1hr at RT. Finally the slides were rinsed 3 times (5–10 minutes) in PBS + 0.1% Triton X-100 (including DAPI in the final wash, and then rinsed twice in PBS) and mounted. Slides were captured on the confocal using Tilescan and Zstack on a 63x objective and merged with FIJI.

### Co-immunoprecipitation

20 E14.5 hearts, and 30 E12.5 hearts, were dissected from WT embryos in cold PBS and flash frozen in liquid nitrogen before being digested in RIPA buffer (50 mM Tris pH 7.4, 150 mM NaCl, 1% NP-40, 1 mM EDTA, 50mM NaF, 0.5% deoxycholic acid, sodium orthovanate 20 nM, anti-protease cocktail 1X, 1 mM PMSF). Sequential syringes with gradually smaller needles (19G, 23G, 25G) were used to help the lysis. Magnetic beads coated with sheep anti-mouse IgG (AB 11201D) were incubated overnight at 4°C with either the supernatant recovered from HIRA hybridomas (WC15) or the mouse IgG1k monoclonal isotype control antibody (AB 18447, lot GR 53099–6). The beads were then washed 3x with the previous RIPA buffer the next day. After the final wash, the protein lysate was incubated with the beads overnight rolling at 4°C. 100 μl of 1 ml was saved and stored at -80°C to load as the input. The next day the beads were washed in RIPA and resuspended in Laemli buffer (3X Laemli: 120 mM Tris pH6.8, 3% SDS, 5% Glycerol, 0.01% Bromophenol, 1.5% β-mercaptoethanol) and boiled for 15min to separate the beads from the antibodies and proteins. Pre-cast gradient gels (Mini-PROTEAN TGX 4%-15%, Biorad 456–1083) were used. 28 μl was loaded per well. The proteins were then transferred onto a PVDF membrane (Biorad 162–0177) using wet transfer. The membrane was blocked with 5% milk-TBST 1X (0.5% tween) for 1 hour at RT, washed 3 times with TBST then incubated with 1/100 of anti-WHSC1 (Atlas HPA015801), or 1/4 of WC119 hybridoma supernatant in 1% milk- TBST 1X overnight at 4°C. Next the membrane was washed 3 times with TBST, incubated with a secondary antibody (Amersham NA93310V) for 45 min at RT then washed 3 times with TBST and finally revealed with ECL (Amersham RPN2209, 28906837) with the appropriate secondary antibody (Amersham NA93340V or NA93310V). The hybridomas WC15 and WC119 were kindly given by Peter Adams.

### Database Deposition

The RNAseq data is deposited at the Gene Expression Omnibus database with accession GSE79937, and ChIPseq data with accession GSE79826.

## Results

### HIRA is required in the developing heart

*Hira* is ubiquitously expressed from E8.5 during mouse embryogenesis [15]. Consistent with this, we detected expression throughout the embryonic heart at E13.5 by using the *β-galactosidase* cassette present in the preconditional *Hira* allele (S1A Fig). We then generated the *Hira* conditional allele using a FLPase transgenic cross (S1B Fig). This *Hira* conditional allele has been previously used in the literature [9], and when combined with an ubiquitous *ActinCre* driver, we observed the same gastrulation phenotypes as in our constitutive *Hira* null embryos (data not shown) [10]. Using our existing constitutive *Hira* null allele (*Hira*^*-*^), we bred *Mesp1Cre* to *Hira*^*-/+*^ mice to generate *Mesp1Cre;Hira*^*-/+*^ mice. Next we mated *Mesp1Cre*;*Hira*^-/+^ with *Hira*^*fl/fl*^ mice to examine the role of HIRA in cardiac progenitors (the single null allele was included so only a conditional allele would be recombined, maximizing Cre-mediated production of *Mesp1Cre* lineage *Hira* null cells). We validated the recombination by PCR (S1C Fig), lack of exon 4 representation in the RNAseq (S1D Fig), and HIRA expression by western blotting (S1E Fig). Of 4 litters born, we did not identify any live *Mesp1Cre;Hira*^*-/fl*^ pups (Table 1), indicating that cardiogenic mesodermal ablation of *Hira* from E6.5 is embryonically lethal.

**Table 1:** Embryonic phenotype resulting from the conditional ablation of *Hira*.

	*Mesp1Cre;Hira*^*-/fl*^				*Nkx2*.*5Cre;Hira*^*-/fl*^	*Mef2cCre;Hira*^*-/fl*^	*Tie2Cre;Hira*^*-/fl*^	*Wnt1Cre;Hira*^*-/fl*^
STAGE	E10.5	E12.5	E13.5	E15.5	E15.5	ADULT	E15.5	E18.5
VSD	N/A	N/A	N/A	12/12	5/9	N/A	0/5	0/10
ASD	N/A	N/A	N/A	10/12	4/9	N/A	0/5	0/10
Thin ventricular wall	0/4	0/9	0/12	3/12	0/9	N/A	0/5	0/10
Constricted PT	0/4	0/9	0/12	1/12	3/9	N/A	0/5	0/10
Oedema	0/4	0/9	3/12	12/12	0/9	N/A	0/5	0/10
Haemorrhage	0/4	0/9	6/12	12/12	0/9	N/A	0/5	0/10
Exencephaly	0/4	1/9	0/12	0/12	0/9	N/A	0/5	0/10
**VIABLE by P10**	**NO (0/12)**				**YES (1/9)**	**YES (4/4)**	**YES (3/6)**	**NO (0/40)**

Number of embryos observed for the indicated phenotype at the indicated stage related to the total number of embryos collected. N/A indicates none observed. The number of collected and thus viable embryos are also indicated at 10 days post-birth (P10) related to the number of expected embryos.

**Fig 1:**
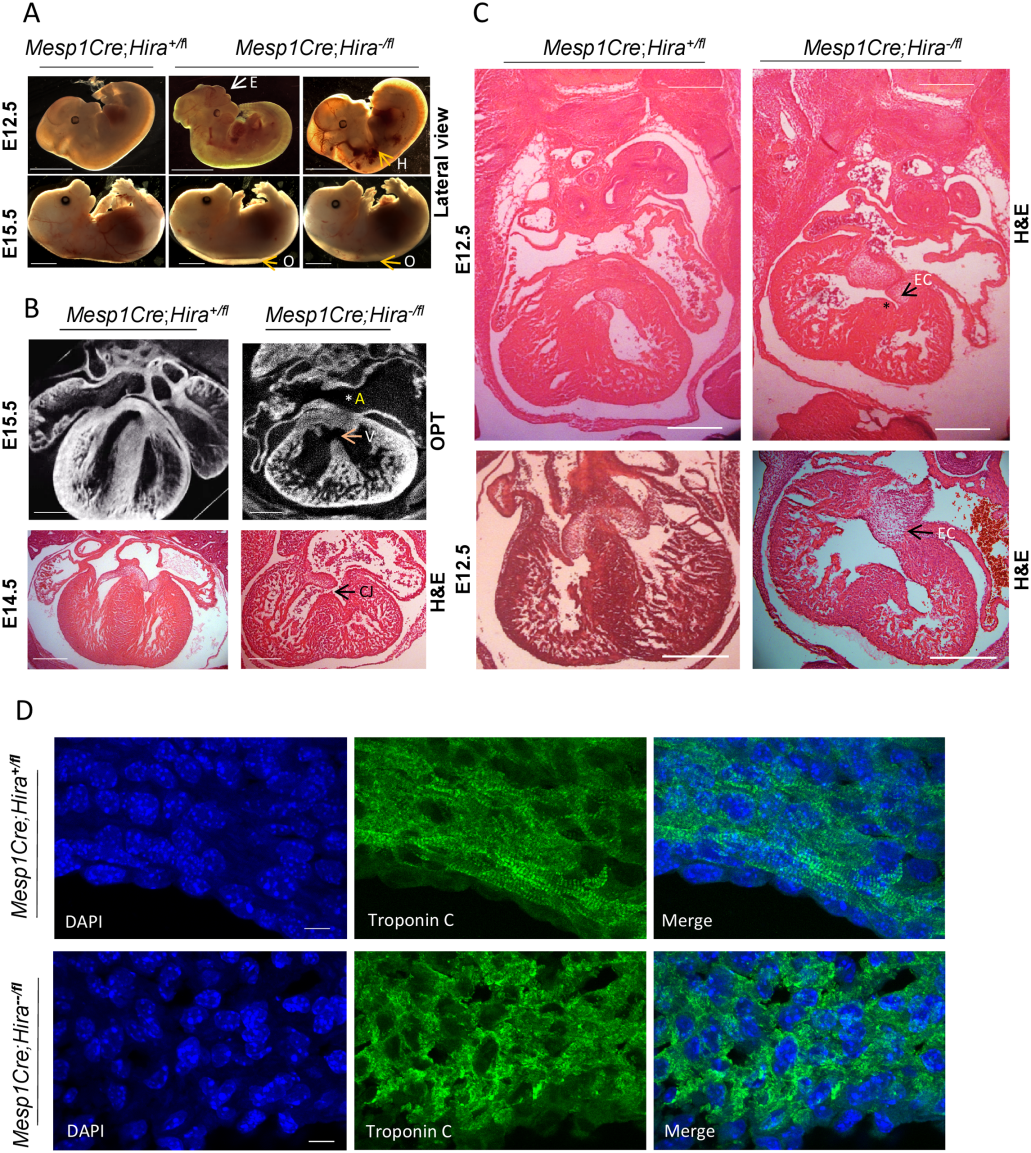
HIRA is required in the developing heart. **A.** Lateral view of littermate embryos with the indicated genotype. *Mesp1Cre*;*Hira*^*-/fl*^ embryos had a low penetrance external phenotype at E12.5: exencephaly (E) and light haemorrhage (H) are indicated in the mutants. At E15.5, all mutants showed severe oedema (O) as indicated. **B.** Transverse OPT reconstructions followed by virtual reslicing of E15.5 embryo trunks with the indicated genotype. VSD (V) and ASD (A) are indicated in the mutants. H&E of transverse sections of E14.5 embryos with the indicated genotype also showed a common atrioventricular junction (CJ) in the mutants as indicated. **C**. H&E staining of transverse sections from E12.5 embryos reveals a disruption of the endocardial cushion (EC) fusion (two controls and two littermate mutants shown). The muscular septum is deficient (*, top) and the relatively flat rather than crescentic cushion shape in the mutant are indicated (arrows). *Scale bars represent 2mm* **(A)**, *0*.*5mm* **(B-C)**. **D**. Transverse sections of E12.5 embryonic hearts of the indicated genotype immunostained with DAPI and Troponin C captured on confocal showing disrupted sarcomeric structure in the mutant ventricular free wall. *Scale bar*: *10μm*.

We detected a small proportion of exencephaly and haemorrhage at E12.5 (Fig 1A, Table 1). All *Mesp1*Cre*;Hira*^-/fl^ embryos presented with a severe oedema at E15.5 (Fig 1A, Table 1). We observed a fully penetrant VSD in the heart of all mutants (n = 12); whilst the muscular ventricular septum completely separated the two ventricles in their littermate WT embryos at E15.5. Some embryos had atrioventricular septal defects with a common atrioventricular junction (Fig 1B, S1 Video). The large interventricular communication observed in *Mesp1*Cre*;Hira*^*-/fl*^ embryos is not likely compatible with life during the late stage of embryogenesis [16]. In 83% of mutants, we detected a deficiency of the flap valve of the oval fossa, a derivative of the primary atrial septum, resulting in an ASD. None was observed in their WT littermates (Fig 1B) (n = 10). Haematoxylin and Eosin (H&E) staining revealed that some E12.5 *Mesp1*Cre*;Hira*^-/fl^ embryos displayed abnormally shaped atrioventricular cushions, whilst mesenchymal tissue normally swells in the atrioventricular canal as a result of EMT in their WT littermates (Fig 1C). In normal development, the rightward tubercles of the atrioventricular cushions form the membranous septum [17, 18]. We have observed deficiency of the muscular part of the ventricular septum, since the point of contact between the septum and the atrioventricular cushions was mispositioned in the mutants compared to controls (Fig 1C). Furthermore, cushions were crescent shaped in the wild type but straighter in the mutants (Fig 1C). Thus, HIRA is required for normal cardiovascular development.

### HIRA is required in the *Nkx*2.5, but not in the *Mef2c* or *Wnt1* lineages

**Fig 2:**
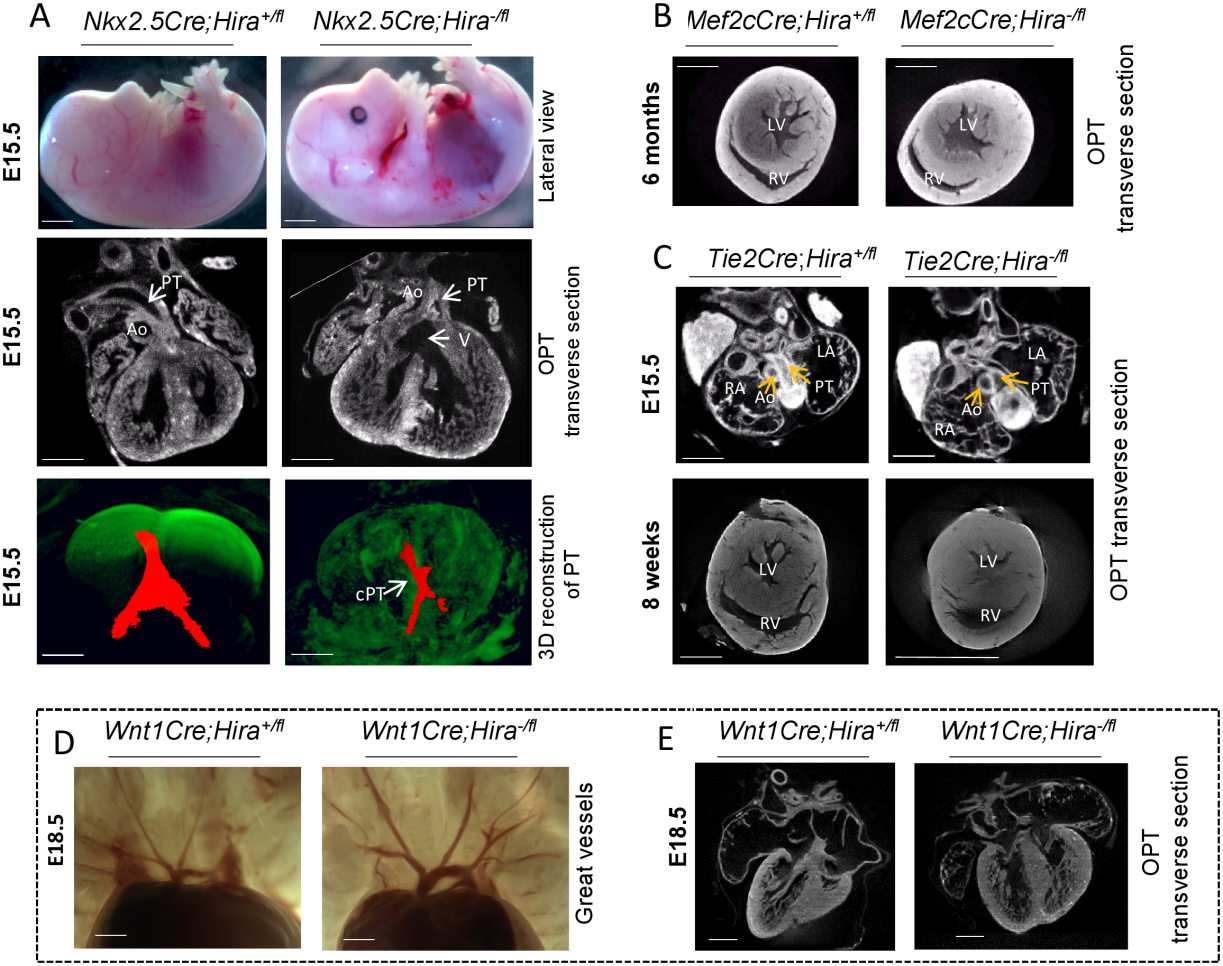
*Hira* requirements in distinct lineages contributing to the heart and vessels. **A.** E15.5 *Nkx2*.*5Cre*;*Hira*^-*/fl*^ embryos and their littermate control embryos were examined as indicated. Lateral view of *Nkx2*.*5Cre*;*Hira*^*-/fl*^ mutants revealed a normal external appearance. OPT transverse section revealed a VSD (V) and an overriding of the aortic root of the muscular septum in as well as a constricted pulmonary trunk PT (cPT) in some mutants by 3D reconstruction. **B&C.** OPT transverse section of the heart of a 6 months old *Mef2cCre;Hira*^*-/fl*^ (right/left ventricle: RV/LV) **(B)** or of *Tie2Cre*;*Hira*^*-/fl*^ embryo and adult **(C)** with their respective littermate control. Mutant embryos did not show any defect and their adult heart only showed proportional size reduction to the body size. The Aorta (Ao), the PT, and the right and left atrium (RA/LA) are indicated. **D&E.** Great vessel structure **(D)** and OPT transverse section (**E**) on E18.5 *Wnt1Cre*;*Hira*^-/fl^ and their control littermate hearts. No heart defects were observed in these mutants. *Scale bars represent 2mm in whole embryo and 0*.*5mm in transverse sections*.

We then refined the requirement of HIRA in cardiac lineages with *Nkx2*.*5Cre* and *Mef2cCre* drivers. The expression of *Nkx2*.*5Cre* is restricted to cardiomyocytes, cardiac endothelium and pharyngeal endoderm [19, 20], and appears 24 hours later than *Mesp1Cre* expression. We observed that 33% (n = 3) of *Nkx2*.*5Cre*;*Hira*^*-/fl*^ embryos had a hypoplastic pulmonary trunk (PT) (Fig 2A). 56% (n = 5) of mutants displayed a large VSD (n = 5) (Fig 2A) but did not show any sign of oedema. The survival rate of mutant pups was extremely low (1 mutant out of 9 expected) (Table 1).

We next mated *Mef2cCre*;*Hira*^*+/fl*^ with *Hira*^*fl/fl*^ mice to test whether HIRA was required in the SHF. *Mef2c* is expressed from E7.5 in the progenitors of the right ventricle, outflow tract, and ventricular septum. *Mef2cCre* driven ablation of *Hira* had no noticeable effect on development and postnatal life (Table 1 and Fig 2B).

A requirement for *Hira* has been described both in endothelial cells [21] and in neural crest cells [22] (in chick), we tested the role of *Hira* in these lineages using *Tie2Cre* and *Wnt1Cre* drivers, respectively. The *Tie2Cre* mutants were fully viable and had no detectable vessel defects at E15.5 (Fig 2C). Nevertheless, adult mutants were smaller at 8 weeks of age, with hearts smaller but in proportion relative to their reduced body size (Fig 2C), and without apparent structural abnormalities by OPT (n = 3). *Wnt1Cre;Hira*^*-/fl*^ embryos demonstrated a perinatal lethality, not due to a cardiac malformation since they presented with a normal heart structure and no apparent vessel malformations at E18.5 (Fig 2D & 2E).

### Absence of HIRA dysregulates cardiac gene expression

**Fig 3:**
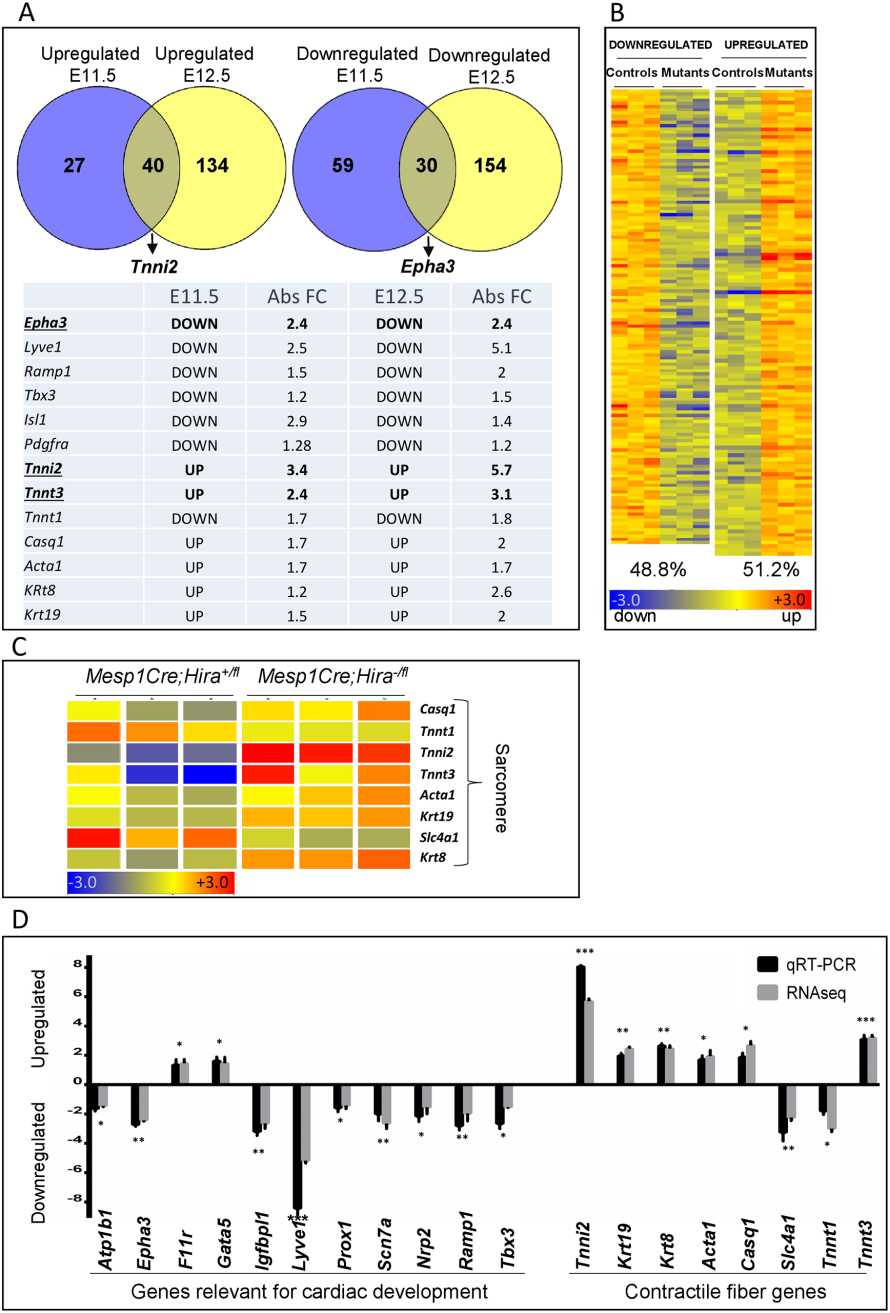
HIRA is required for the regulation of a subset of genes expressed during cardiac morphogenesis. **A.** Venn diagram indicating the number of genes whose expression is up or downregulated in *Mesp1Cre*;*Hira*^*-/fl*^ compared to *Mesp1Cre*;*Hira*^*+/fl*^ hearts at E11.5 and E12.5. The table indicates the level of fold change observed at E11.5 and at E12.5 on a subset of genes. **B.** Heatmap displaying the differential gene expression in mutant *(Mesp1Cre*;*Hira*^*-/fl*^) vs control *(Mesp1Cre*;*Hira*^*+/fl*^) in triplicates at E12.5. **C.** Heatmap of the gene ontology analysis revealed a trend in upregulation of sarcomeric contractile fibre genes (aside from *Tnnt1 and Slc4a1* which were downregulated). **D.** qRT-PCR and RNASeq of the indicated genes within E12.5 hearts displayed as the fold induction in the mutants compared to their WT littermates (n = 3). Unpaired t-test: p<0.05 *, p<0.01 **, p<0.001 ***.

The full penetrance of *Mesp1Cre Hira-*conditional mutants allowed us to investigate the transcriptional changes underlying the cardiovascular defects observed in the absence of HIRA. We chose the E11.5 and E12.5 stages as they were prior to the appearance of the major phenotypes. At E11.5 and E12.5 stages there were 156 and 360 coding transcripts respectively with significantly altered expression in the mutant hearts (Mann Whitney unpaired test, Benjamini Hochberg FDR, *p* ≤ 0.05, FC ≥ 1.5) (Fig 3A, S1 and S2 Tables), with no trend towards up- or down-regulation of global transcription (48.8% down and 51.2% up; Fig 3B). We performed a gene ontology (GO) analysis with the differentially expressed genes and observed a link with myocyte contractility (S3 Table). Four GO terms included the same eight genes related to contractility and sarcomeric structure (Fig 3C), including the most up-regulated gene *Tnni2*. We next used quantitative real-time PCR (qRT-PCR) to validate expression differences for 11 changed genes that are relevant to cardiac development, and the eight genes related to cardiac contractility (Fig 3D).

**Fig 4:**
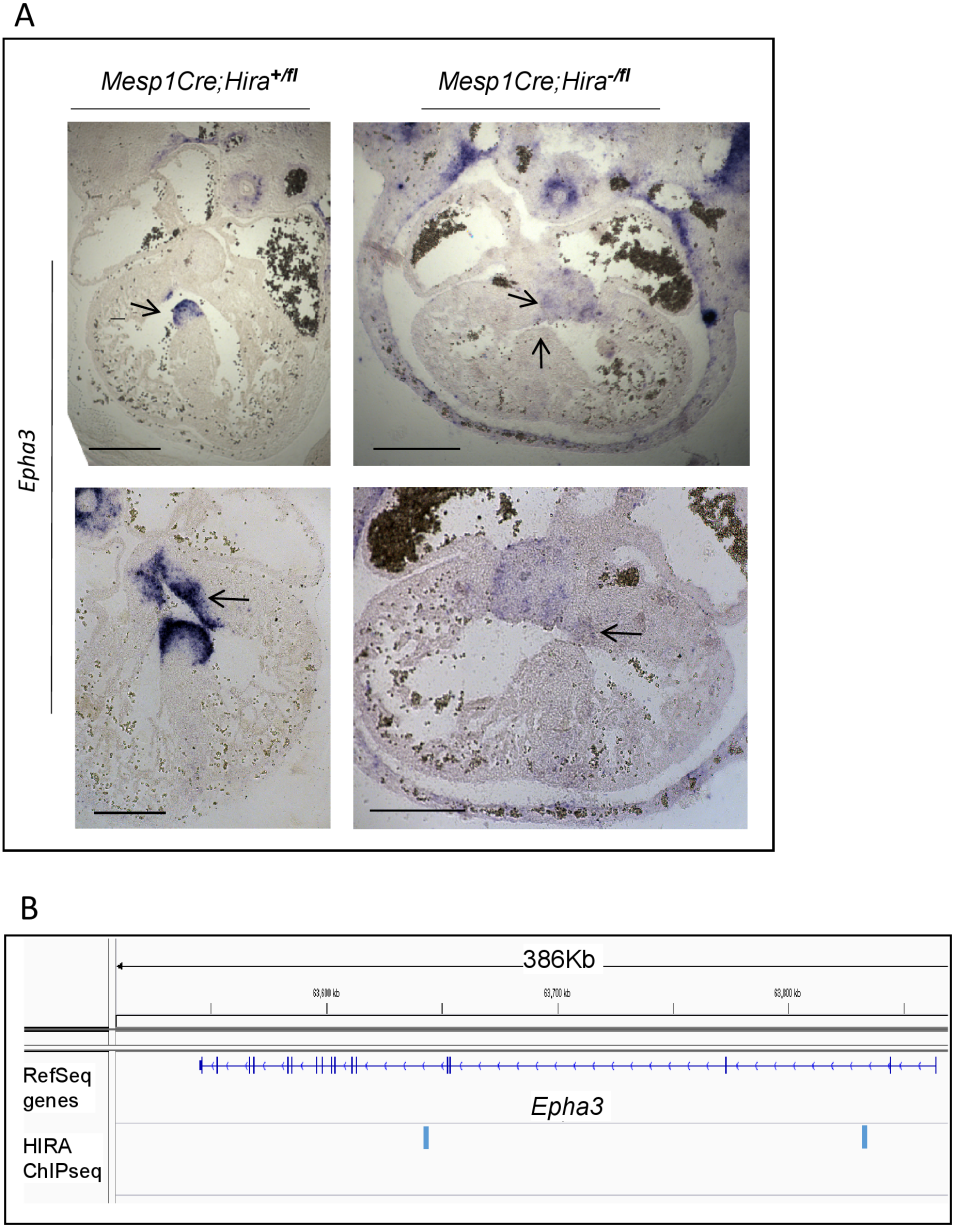
*Epha3* is downregulated when HIRA is conditionally deleted, and HIRA binds to the gene body of *Epha3*. **A**. *In situ* hybridization of *Epha3* on transverse sections of E12.5 embryonic hearts with the indicated genotypes (n = 2). *Hira*-conditional mutant hearts displayed low expression of *Epha3* in both the membranous portion of the septum (upper panels) and the endocardial cushions (lower panels) compared to littermate controls as indicated by a black arrow. *Scale bar*: *100μm* **B.** IGV profile showing two HIRA enriched loci in intronic regions of *Epha3*.

**Fig 5:**
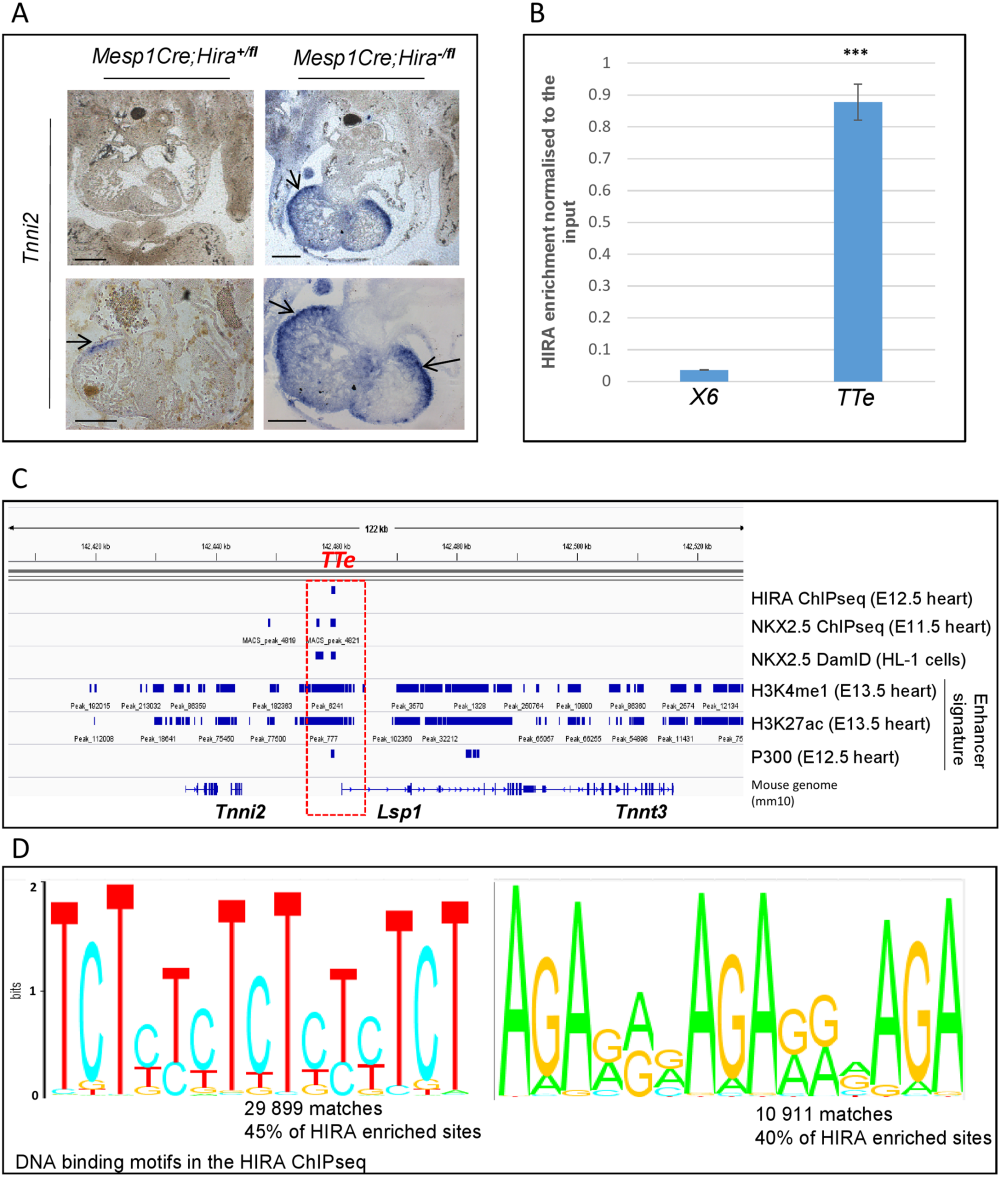
HIRA is enriched in the vicinity of *Tnni2*/*Tnnt3*, which is upregulated in conditionally deleted *Hira* mutants, and binds GAGA rich DNA. **A.** *In situ* hybridization *Tnni2* on transverse sections of E12.5 embryos with the indicated genotype, revealed an abnormally increased expression of *Tnni2* particularly in the ventricular free wall of the mutant hearts (arrows). *Scale bar*: *100μm* **B**. qChIP of the HIRA enrichment at the *TTe* locus. An intergenic region on chromosome 6 was used as a negative control. Data is normalized to the input (= 100) and is representative of 2 independent experiments. Errors bars represent the min/max from technical duplicates. Unpaired t-test: p<0.001 *** **C.** IGV profile showing the HIRA ChIP seq peak present at the *Tnni2/Lsp1/Tnnt3 (TTe*) site (situated 17Kb downstream of *Tnni2* and 34Kb upstream of *Tnnt3)*, co-localizing with NKX2.5 ChIPseq peaks (E11.5 heart [11]), NKX2.5 DamID peaks (HL-1 cell line [12]), and the active enhancer marks H3K4me1 and H3K27Ac ChIPseq peaks on WT E13.5 hearts (encode database ENCSR663VWL) and P300 (WT E12.5 heart [23]). **D.** The Matrix-based nucleotide profiles display here the motifs that HIRA binds most frequently. 45% of HIRA enriched sites contained a GAGA/TCTC motif.

We examined selected genes known for their role in cardiac development and displaying the highest expression change in our mutants in both E11.5 and E12.5 mutants (Fig 3A). The expression of *Epha3*, a receptor tyrosine kinase required for EMT in the atrioventricular cushions [18], was downregulated of 2.7 fold in the heart of the mutants compared to their control littermates (Fig 3D). In agreement, *in situ* hybridization (ISH) revealed that *Epha3* expression was greatly diminished in the cushions and the membranous part of the ventricular septum in the mutants compared to their control littermates (Fig 4A). We also found that *Tnni2*, a fast twitch skeletal muscle gene, was the most upregulated gene in the mutant hearts by both RNA-seq (5.9 fold) and qRT-PCR (7 fold) (Fig 3D). ISH confirmed *Tnni2* overexpression in the mutants (Fig 5A), suggesting heart contractility could be affected by alteration of sarcomeric components. Indeed, staining with Troponin C on transverse sections to examine sarcomeric organisation demonstrated a lack of typical parallel organisation of the filament structures in the mutants (Fig 1D). There were no significant transcription changes in *Hira*^+/-^ hearts at E12.5 (S4 Fig).

### HIRA is enriched in the vicinity of the *Tnni2*/*Tnnt3* gene loci

To assess whether HIRA was directly regulating gene expression, we performed a HIRA ChIPseq of WT E12.5 hearts. We found 6625 peaks using Model-based Analysis for ChIPseq (MACS) analysis (p≤10^−4^) which mostly covered distal intergenic regions of the genome (74%) and introns (22.5%) (S2A Fig). Interestingly, 45% of the peaks contained the consensus motif GAGAGAGA (Fig 5D) that in *Drosophila melanogaster* is known to bind the GAGA factor.

**Fig 6:**
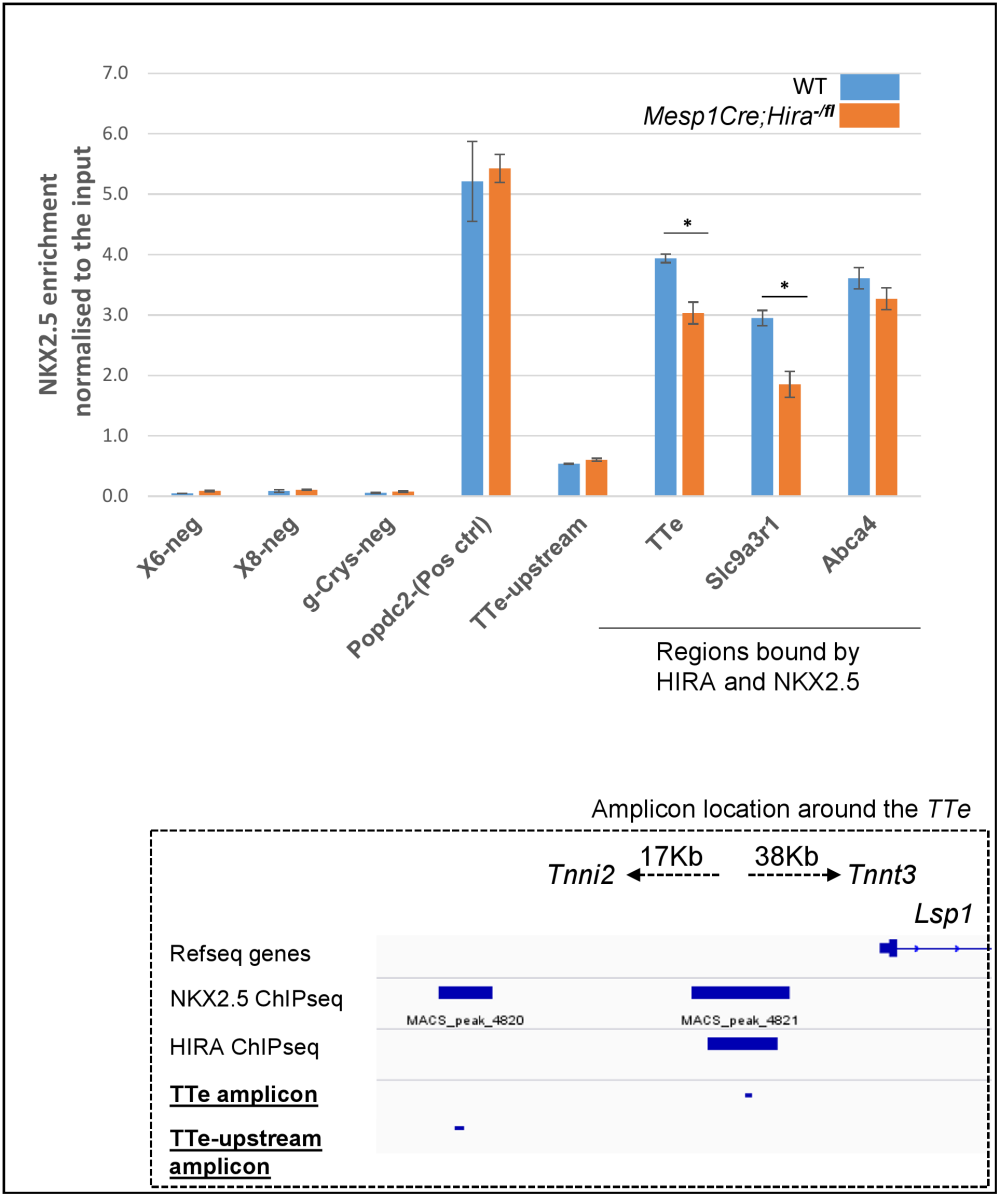
NKX2.5 shows diminished binding at the *TTe* locus in conditionally HIRA depleted hearts. NKX2.5 qChIP on 20 WT and *Mesp1Cre*;*Hira*^*-/fl*^ E12.5 hearts. Enrichment at the *TTe* was confirmed and found to be reduced in mutant hearts. *Slc9a3r1* and *Abca4* were enriched for both HIRA and NKX2.5 in WT hearts and displayed an increased expression in both *Hira* and *Nkx2*.*5* mutant hearts. *SLc9a3r1*, but not *Abca4*, showed a significantly reduced binding of NKX2.5 in the absence of HIRA. Three different negative regions were tested. A region known to be enriched for NKX2.5, but not HIRA, binding (*Popdc2)* was used as a NKX2.5 qChIp positive control. Data is normalized to the input (= 100). Errors bars represent the min/max from technical duplicates. Unpaired t-test: p<0.05 *. Subpanel represents the different ChIPseq peaks along with the PCR amplicons used in the different qChIp at *TTe*, and at a locus bound by NKX2.5, but not by HIRA.

Two significant HIRA-bound regions were identified in intronic regions of *Epha3* (Fig 4B), of which one contained a GAGA motif. One GAGA motif was observed within a significant HIRA-bound region 17Kb downstream of *Tnni2*, 38Kb upstream of *Tnnt3*, and 1 kb of *Lsp1* (Fig 5B). We subsequently refer to this peak as the *Tnni2-Tnnt3-enh (TTe)* site (see below). The expression of both *Tnni2* and *Tnnt3* was strongly upregulated in our mutant model however, we did not observe any dysregulation of *Lsp1* expression. This is strikingly similar to what is observed in *Nkx2*.*5* hypomorphs, which show *Tnni2/Tnnt3*, but not *Lsp1*, overexpression in E11.5 mutant versus wild type hearts [11] (S2B Fig). Moreover, it has been shown that the *Tnni2* and *Tnnt3* genes are directly repressed by NKX2.5 via direct binding to the *TTe* site (E11.5 hearts), and that the *TTe* sequence can direct reporter expression in the HL-1 (cardiomyocyte) cell line [11]. We found a 25-fold enrichment of HIRA at the *TTe* compared to a negative intergenic control region by qChIP (Fig 5B). We next examined active enhancer marks by overlapping HIRA E12.5 ChIPseq peaks with the histone modification H3K4me1and H3K27Ac ChIPseq peaks at E13.5, as well as P300 in embryonic hearts at E12.5 [23] at the *TTe* (Fig 5C) and genome wide (S2C Fig). Of the 78 sites containing overlaps of HIRA and NKX2.5, 63 (80.7%) overlapped with both H3K4me1 and H3K27Ac, including *TTe*. We interrogated these histone marks at the *TTe* in ESCs differentiated to cardiomyocytes and in their originating ESCs [24] (S3 Fig). The *TTe* locus became enriched for H3K4me1 and H3K27Ac in differentiated cardiomyocytes compared to ESCs, in agreement with the *TTe*-reporter data indicating that *TTe* is an active enhancer in the heart [11]. We next tested whether absence of HIRA affected NKX2.5 occupancy at the *TTe* by performing a NKX2.5 qChIP. At E12.5, NKX2.5 binding was moderately but significantly reduced to 75% (Fig 6) in *Mesp1Cre;Hira*^*fl/-*^ hearts compared to WT hearts. In addition to *Tnni2* and *Tnnt3*, we examined three other genes dysregulated in both *Mesp1Cre;Hira*^*fl/-*^ and *Nkx2*.*5* hypomorphic E11.5 hearts: *Clcnkb*, *Abca4* and *Slc9a3r1*. Similarly to *Tnni2* and *Tnnt3*, all three were upregulated by 2.9, 1.72 and 1.53 fold respectively in *Hira* mutant hearts. A reduced NKX2.5 binding at the *Slc9a3r1* locus was also observed in the hearts of *Hira* conditional null mutants (Fig 6).

**Fig 7:**
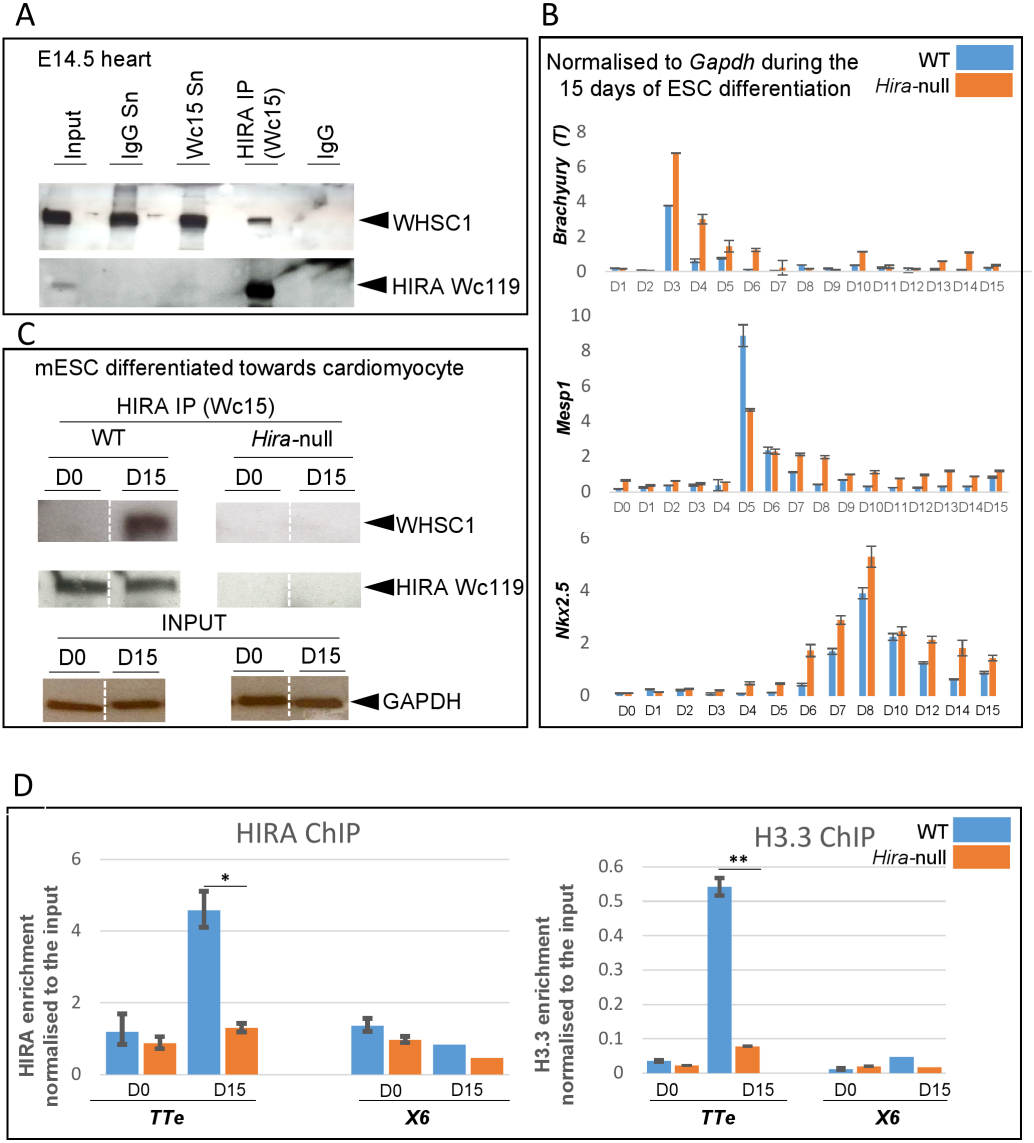
HIRA interacts with WHSC1 and binds to the *TTe* in the cardiac lineage and heart, and is required for differentiation-associated H3.3 enrichment at this locus. **A.** 20 E14.5 hearts from WT embryos were isolated, pooled and immunoprecipitated with HIRA Wc15 antibody and immunoblotted with WHSC1. Presence of HIRA in the IP was verified using Wc119 antibody. **B.** Mouse ESCs (control and *Hira*^*-/-*^ ES cells) were differentiated towards cardiomyocytes over 15 days in LIF free medium following the hanging drop method, and were subjected to RNA and protein isolation. qRT-PCR of *Brachyury (T)*, *Mesp1 and Nkx2*.*5* over the 15 days are presented and demonstrate sequential expression of the early mesoderm, cardiogenic mesoderm, and cardiac markers respectively. Data are shown as levels of mRNA normalized to *Gapdh* and are representative of five independent experiments. Errors bars represent the standard deviation of technical triplicates. **C.** Protein isolated from undifferentiated ESCs and 15 days differentiated control and *Hira*^*-/-*^ ESCs were immunoprecipitated with anti-HIRA Wc15 antibody and immunoblotted with anti-WHSC1 antibody. The results shown are all from the same western membrane but separated lanes are brought into proximity and separated by dashed bars. GAPDH is used as a control for input. **D.** HIRA and H3.3 qChIP on differentiating ESCs shows an enrichment of HIRA at the *TTe* at day 15. H3.3 enrichment at the *TTe* increases significantly in WT but not in *Hira* null ESCs following this differentiation. X6 is an intergenic negative control region. Unpaired t-test was used: p<0.05 *, p<0.01 **.

As NKX2.5 interacts with the WHSC1 histone methyltransferase to repress target genes during heart development [25], and in HeLa cells HIRA interacts with WHSC1 [26], we tested whether there was a HIRA-WHSC1 interaction in mouse embryonic hearts using co-immunoprecipitation. We detected an interaction between HIRA and WHSC1 at E12.5 (S5 Fig) and at E14.5 (Fig 7A).

We then used previously reported WT and *Hira-*null ESCs carrying an HA-tag knock in of H3.3 [4] to investigate potential interactions during the early stages of cardiac differentiation. The cells were differentiated for fifteen days using the aggregation method in order to obtain cardiogenic mesoderm and primitive cardiomyocytes. qRT-PCR was performed to assess the sequential gene expression of specific mesodermal and cardiac markers. *Brachyury*, *Mesp1* and *Nkx2*.*5 were* expressed sequentially at days 3, 5 and 8 of differentiation respectively (Fig 7B), and spontaneously beating cardiomyocytes were observed (S1 Video). At day 15 of differentiation towards cardiomyocytes, the HIRA-WHSC1 interaction was observed (Fig 7C).

### HIRA regulates H3.3 deposition at the *TTe* in differentiated embryonic stem cells

Using the same ESC culture conditions as above, we then tested HIRA and H3.3-HA enrichments at the *TTe* by qChIP at D0 and D15 of differentiation. We found enrichment of HIRA and H3.3 at the *TTe* in WT, but not *Hira* null, differentiated cells (Fig 7D).

## Discussion

Modification of chromatin is well established as an important mechanism of genetic regulation and is usually associated with covalent modification of histones or deposition of variant histones, or both [27]. In this work we examine the role of the replication independent histone chaperone component HIRA, a known modulator of gene expression [28]. Previous work suggested HIRA acts during vascular EC development [21] and in cardiac neural crest cells [29]. *Hira* knockout embryos had abnormal cardiac structure and function [10], but as these embryos were delayed with yolk sac vascular anomalies it was not known whether these defects were secondary events.

We successfully bypassed early lethality of *Hira*^-/-^ embryos using a *Hira* conditional allele, demonstrating HIRA plays a crucial role in the cardiogenic mesoderm. At E15.5 all *Mesp1Cre;Hira*^*-/fl*^ embryos presented with whole body oedema and large VSDs, the majority of which were accompanied by ASDs, defects likely incompatible with life. No live born mutants of this genotype were recovered. *Nkx2*.*5Cre* is mainly expressed in ventricular cardiomyocytes of the FHF and SHF, although the extent and degree of expression in the SHF appears to be reporter gene and genetic background dependent [30, 31]. In *Nkx2*.*5Cre;Hira*^*-/fl*^ embryos we detected an overriding aorta, a VSD, and a constriction of the PT in some mutants. The lack of phenotype in *Mef2c* conditional knockouts suggests that *Hira* is required in the FHF and ablation in the SHF has no effect or acts to exacerbate the phenotype of FHF mutants. We note that the *Nkx2*.*5Cre* used here is a knock-in allele and thus there is the potential for interaction between hemizygous levels of NKX2.5 and complete absence of HIRA. However, we did not observe any phenotype in *Nkx2*.*5Cre;Hira*^*+/fl*^ embryos.

As the *Mesp1Cre* lineage includes vascular ECs we investigated whether *Hira* was required for vessel development within this population using *Tie2Cre*, which is active from E7.5 [32]. Moreover, HIRA has been previously shown to mediate the response to angiogenic signals by upregulating *Vegfr1* in yolk sac ECs [21]. The authors of this work concluded that HIRA was required for growth of new blood vessels. However, we found that *Tie2Cre;Hira*^*-/fl*^ mutants suffered only from partially penetrant proportionate growth reduction, with no embryonic lethality despite efficient recombination of the conditional allele [21]. The absence of vessel defects and embryo oedema suggests that the oedema observed in *Mesp1Cre* mutants is not secondary to an EC defect and is therefore more likely due to the septal defects and any disturbance of contractility secondary to alterations in troponin gene expression (see below).

Experiments in chick, using antisense morpholinos directed against *Hira*, suggested that *Hira* may play a role in the cardiac neural crest [29] and is required for outflow tract septation. The absence of cardiac and great vessel abnormalities in the *Wnt1Cre*;*Hira*^*-/fl*^ embryos up to E18.5 argues against an autonomous role of HIRA in cNCCs during cardiovascular development. The perinatal death that was observed in *Wnt1Cre*;*Hira*^*-/fl*^ mutants could rather be the result of a neurological problem or a cleft palate defect interfering with swallowing [33], since *Wnt1Cre* is expressed in craniofacial NCCs. In considering these results it is worth noting the recent discussion of the fact that various murine mutations of genes expressed in NCCs produce different phenotypes to those of other vertebrates [34].

We next asked how absence of HIRA affected the gene expression profile in the developing heart. We chose to analyse *Mesp1* conditional knockouts since this was a setting with a 100% penetrant phenotype (by E15.5). In total, less than 2% of the genes expressed in the heart at E12.5 were significantly upregulated or downregulated. Several genes critical for heart development were unaffected by the ablation of *Hira*, e.g. *Myh6/7*, *Gata4*, *Nkx2*.*5* and *Tbx5*, implying that HIRA has a specific effect on transcription at a small number of target genes. In order to understand the relevant molecular mechanism we performed a HIRA ChIPseq on E12.5 hearts. We detected HIRA enrichment at 6625 loci mapped to 2515 genes, with mapping defined as the presence of a peak in the gene body and/or within 5Kb upstream of the TSS and downstream of TES. There were 47 such genes that had a dysregulated expression in the absence of HIRA by RNA-seq, although this was not a statistically significant increase over chance. Further work, such as chromatin conformation capture, would be required to corroborate peak-gene relationships. It should also be borne in mind that, as for the *TTe* site discussed below, relevant enhancer binding sites may be >5kb from a target gene. Nevertheless, we reason HIRA acts directly at a relatively small subset of genes, and indirectly modulates expression via more general effects on chromatin structure acting over longer ranges. This is consistent with our finding that just over 7.5% of HIRA peaks at E12.5 were found to overlap with both H3K4me1 and H3K27Ac marks (at E13.5, the closest stage available).

Examination of HIRA binding sites for over representation of sequences that might be responsible for HIRA recruitment revealed that the most frequent sequence had a core consensus site GAGAGAGA, which is equivalent to that identified as the binding motif for the *Drosophila melanogaster* transcriptional regulator GAGA factor [35]. GAGA factor interacts with FACT, and intriguingly GAGA/FACT associates with Drosophila HIRA. Together, these proteins direct H3.3 replacement to establish chromatin boundaries e.g. at HOX loci [36].

Interestingly, HIRA enrichment at the *TTe* locus in the wild type situation correlates with an upregulation of cardiac *Tnni2* (7 fold) and *Tnnt3* (3.2 fold) following conditional ablation in the cardiogenic lineage. The encoded proteins are classified as fast skeletal muscle troponins [37]. The troponin complex binds calcium ions, and through its association with actin and tropomyosin is involved in the regulation of striated muscle contraction. While various troponins are expressed in developing heart at E12.5 [38], skeletal troponins have low expression in cardiac muscle, and upregulation of *Tnnt3* has been associated with reduced cardiac contractility [39]. In a *Notch1* gain of function mutant, structural defects of the myocardium were attributed to elevated expression of *Tnni2* [40]. GO analysis of the RNAseq revealed that several sarcomeric genes (also under the GO term myofibril) were dysregulated. *Tnni2*, *Tnnt3*, *Casq1*, *Acta1*, *Krt8*, *Krt19* [41] were upregulated and the anion exchanger *Slc4a1* and *Tnnt1* were downregulated. The disorganised sarcomeric structure we observed within mutant embryonic cardiomyocytes likely contributes to diminished efficiency of contraction [42]. The mouse heart starts beating at E8.5; reduced cardiac output can lead to whole body oedema at E14.5 [43]. Thus, the haemorrhage and oedema we observed at E15.5 could be a consequence of both morphological abnormalities (septal defects) and compromised cardiomyocyte function.

The dysregulation of *Tnni2/Tnnt3* was investigated further in the light of recently published work demonstrating that both in the HL-1 cardiomyocyte cell line (DamID screening) and E11.5 whole hearts (ChIPseq) NKX2.5 bound a sequence 17kb downstream of *Tnni2* and 38kb upstream of *Tnnt3* coincident with our HIRA binding site (Fig 5C) [11, 12]. In hearts of E11.5 and E14.5 embryos with a hypomorphic allele of *Nkx2*.*5* there was upregulation of *Tnni2* and *Tnnt3* [11], recapitulating what we observed in *Hira* conditionals, raising the possibility that HIRA and NKX2.5 might co-regulate this locus. Absence of HIRA resulted in a diminution of NKX2.5 binding at the *TTe*.

The literature suggested a further potential mechanism whereby NKX2.5- and HIRA-regulation might converge. In fibroblasts HIRA was previously described to interact with the histone-lysine N-methyltransferase WHSC1, regulating the H3.3 deposition and H3K36me3 marks at interferon-regulated genes [26]. *WHSC1* is a gene haploinsufficient in the Wolf-Hirschhorn syndrome, a human birth defect where congenital heart defects, usually atrial and/or ventricular septal defects, occur in 30–45% of cases [44]. *Whsc1-*null mice also have atrial and ventricular septal defects [25]. Whereas no *Nkx2*.*5*^*+/-*^ or *Whsc1*^*+/-*^ mice had septal defects, such abnormalities were found in one third of double heterozygotes [25]. In support of such a connection we found HIRA and WHSC1 co-immunoprecipitated from embryonic hearts and cardiomyocytes. It will therefore be interesting to investigate whether WHSC1 and HIRA co-regulate *Tnni2* and other targets during heart development analysing compound mutants and histone modifications in such mice. Both *NKX2*.*5* and *WHSC1* are implicated in human genetic haploinsufficiency resulting in congenital heart defect. However, to our knowledge, no mutations of *HIRA* have been described in exome sequencing analyses of patients with congenital heart defect, although one study of a Chinese cohort discovered a genetic association between a variant in the 3’ UTR region of HIRA and the diagnosis of tetralogy of Fallot [45].

As HIRA is known to deposit the H3.3 variant within chromatin we examined H3.3 levels at the *TTe* site in the presence and absence of HIRA. We utilized a cell line model akin to that used by Wamstad and colleagues [24] to examine H3.3 deposition in ESC-derived cardiomyocytes, making use of an HA-knockin to H3.3. We confirmed HIRA enrichment at the *TTe* in differentiated ESCs (equivalent to Wamstad stages 3 and 4 based upon marker expression and the presence of beating cardiomyocytes) versus undifferentiated ESCs, and observed a 7 fold increase of HIRA-dependent H3.3 deposition at the *TTe*.

We also examined one of the most significantly down-regulated genes at both E11.5 and E12.5 which had a known role in heart development: *Epha3*. *Epha3* is expressed in the atrioventricular cushion and mesenchymal cap of the developing septum primum from E10.5 [18]. Accordingly, we examined expression in E12.5 *Mesp1Cre*;*Hira*^*-/fl*^ mutants hearts and observed a consistent downregulation of *Epha3* in precisely those regions of mutants versus the controls. Interestingly, *Epha3-*null embryos are perinatal lethals with ASD and cardiac failure, and have earlier atrioventricular cushion defects [18]. This mirrors several defects seen at E12.5 in *Mesp1Cre;Hira*^-/fl^ mutant hearts. Thus, we reason diminished expression of *Epha3* is likely to contribute to the septal defects in *Hira* mutants. *Epha3* expression was not significantly altered in *Nkx2*.*5* hypomorphs [11], nor was there evidence of NKX2.5 binding close to *Epha3* in either the DamID [12] or NKX2.5 ChIPseq datasets [11]. There were two HIRA binding regions at *Epha3*, one of them containing the GAGA consensus site. Thus, we anticipate other HIRA partners might be involved in transcription activation at this locus. The NKX2.5-WHSC1 complex is thought to repress targets [25], so the absence of NXK2.5 binding sites at the down-regulated *Epha3* is not unexpected.

In summary, our data provide the first *in vivo* demonstration in vertebrates of a post-gastrulation requirement for the histone chaperone complex protein HIRA: conditional mutagenesis in cardiogenic mesoderm resulting in severe structural defects and evidence of heart failure leading to embryonic lethality. HIRA was shown to bind to the same enhancer of *Tnni2* as the cardiogenic transcription factor NKX2.5, suggesting regulation of specific loci as well as more general effects on chromatin represent an important subset of the pleiotropic functions of HIRA. This work also emphasises HIRA can act in repressive as well as activating situations, as has recently been shown in ESCs and plants [5, 46]. It has previously been shown that the WD repeats and LXXLL motifs are necessary for HIRA-mediated repression in vitro [22]. In ESCs, only a third of enhancer sites bound by HIRA had significantly reduced H3.3 levels in the absence of HIRA [5]. Thus, it will be interesting to determine the role of other H3.3 chaperone associated proteins, such as DAXX, during heart development. Moreover, as a member of a replication independent histone chaperone complex, HIRA might have a particular role in post-mitotic tissues later in life, for instance in the aging of cardiomyocytes and neurones, and in their response to injury.

## Supporting Information

S1 Fig *Hira* expression in the heart and recombination of the conditional allele. **A.** β-Galactosidase assay on E10.5 and E13.5 *Hira*^*Sanger/+*^ and *Hira*^*+/+*^ embryos, either on whole mount or on heart sections as indicated, showing an ubiquitous expression of *Hira*. **B.** Schematic representation of the different *Hira* alleles used in the manuscript. **C.** Recombination of the floxed allele by *Mesp1* driven CRE recombinase was assessed by PCR using DNA from the anterior limb which contains *Mesp1* positive cells, detection of the various alleles is shown. **D.** Recombination was also demonstrated at the mRNA level by the reduced number of reads in RNAseq (22 in control and 3 in mutant). **E.** Reduced protein level of HIRA in *Mesp1Cre*;*Hira*^-/fl^ hearts. This experiment was done by comparing pools of 3 *Hira*^+/fl^ and 3 *Mesp1Cre*;*Hira*^-/fl^ E13.5 hearts. Remaining protein is likely to originate from non-*Mesp1*-expressing cardiac linages (e.g. circulating cells and ingressing neural crest).
(TIFF)

S2 Fig Analysis of HIRA ChIPseq. **A.** Table summarising the genic distribution of HIRA binding sites and the GAGA motifs across the genome. 45% of HIRA peaks were found to have a GAGA motif. **B.** Venn diagram displaying the number of genes upregulated in hearts of E12.5 *Mesp1Cre*;*Hira*^-/fl^ embryos and in E11.5 *Nkx2*.*5* hypomorph hearts [11]. **C.** Venn diagram displaying the number of overlaps between HIRA ChIPseq peaks at E12.5 (this study), enhancer signatures H3K4me1 and H3K27Ac at E13.5 (encode database ENCSR663VWL) and NKX2.5 ChIPseq peaks in E11.5 hearts [11] as indicated. **D. a.** Venn diagram displaying the overlap between genes whose expression is dysregulated in E12.5 *Mesp1Cre*;*Hira*^*-/fl*^ hearts and genes which are enriched for HIRA in WT E12.5 hearts. The enrichment includes any peak in the gene body and/or within 5Kb upstream of the TSS or downstream of TES. **b.** The equation for the calculation of the probability of having 47 genes (x) in common between two independent groups: 2515 (b, HIRA ChIPseq genes) and 360 (a, RNAseq data) in the mouse genome which has approximately 22 000 genes (n). The result of this hypergeometric probability calculation is not significant: *p*(x > = 47) = 0.184.
(TIFF)

S3 Fig Histone enrichments at the *TTe* showing the characteristics of an enhancer in ESC derived cardiomyocytes compared to embryonic stem cells. The enrichment of H3K4me1 and H3K27ac and the lack of repressive H3K27me3 modifications represent a distinct chromatin pattern observed in active enhancers (black box around the *TTe*). The development of this pattern mirrors the upregulation of *Tnni2* seen during the differentiation process and supports the association of the *TTe* enhancer with its expression. Histone ChIpseq in ESC derived cardiomyocytes and ESCs were obtained from Wamstad and colleagues [24] (Gnomex accession numbers 44R and 7R2).
(TIFF)

S4 Fig Gene expression is not significantly affected in Hira^-/+^ embryonic hearts at E12.5. A subset of genes affected in *Mesp1Cre*;*Hira*^*-/fl*^ hearts was quantified in *Hira*^*+/-*^ mutant hearts using real time PCR. No significant changes were detected in *Hira* heterozygotes.
(TIFF)

S5 Fig HIRA interacts with WHSC1 in the heart at E12.5. 30 embryonic hearts from WT embryos were isolated at E12.5, pooled and immunoprecipitated (IP) with anti-HIRA Wc15 antibody then immunoblotted with anti-WHSC1 antibody. Presence of HIRA in the IP was verified using anti-HIRA Wc119 antibody.
(TIFF)

S1 Table Genes identified through RNAseq with a significant decreased expression in *Mesp1Cre;Hira*^*-/fl*^ embryonic hearts at E12.5. (TIFF)

S2 Table Genes identified through RNAseq with a significant increased expression in *Mesp1Cre;Hira*^*-/fl*^ embryonic hearts at E12.5. Presented here are genes with the lowest p-value. The fold change is presented here as an absolute value. Test applied: Mann-Whitney unpaired, Benjamini Hochberg FDR, p ≤ 0.05, FC ≥ 1.5. The complete list can be accessed with at the GEO database under accession number GSE79937.
(TIFF)

S3 Table Gene Ontology terms obtained from RNAseq. GO analysis of the genes significantly dysregulated in *Mesp1Cre;Hira*^*-/fl*^ embryonic hearts that have an over-representation of one or more GO terms that pass the cut-off p-value of 10^−4^. Terms relating to contractility and myofibril structure are highlighted in orange.
(TIFF)

S4 Table Primers used for qRT-PCR. (TIFF)

S5 Table Primers used for qChIP. (TIFF)

S1 Video 1: Transverse reconstruction of an OPT scan of *Mesp1Cre;Hira*^*+/fl*^ and *Mesp1Cre;Hira*^*-/fl*^ embryonic trunks at E15.5 with the VSD indicated. 2: 3D reconstruction of the PT in *Nkx2*.*5Cre;Hira*^*+/fl*^ and *Nkx2*.*5Cre;Hira*^*-/fl*^ embryonic hearts at E15.5. 3: Beating ESCs observed in differentiation experiment.
(7Z) Click here for additional data file.

## Abbreviations

ASD: Atrial Septal Defect
AVSD: Atrioventricular septal defect
ChIP: Chromatin Immunoprecipitation
ChIPseq: ChIP followed by sequencing
ESC: Embryonic stem cell
EC: Endothelial cells
EMT: Endothelial to mesenchymal transition
FHF: First heart field
GO: Gene ontology
H&E: Haematoxylin and Eosin
HUCA: HIRA/UBN1/CABIN1/ASF1a complex
ISH: *In situ* hybridization
cNCC: Cardiac neural crest cell
OFT: Outflow tract
OPT: Optical projection tomography
PT: Pulmonary trunk
qChIP: Quantitative Chromatin Immunoprecipitation
qRT-PCR: Quantitative real-time PCR
RT: Room temperature
SHF: Second heart field
TES: Transcriptional end site
TSS: Transcriptional start site
*TTe*: *Tnni2-Tnnt3* enhancer
VSD: Ventricular septum defect
