# Supplementary figures and images for: HIRA Is Required for Heart Development and Directly Regulates Tnni2 and Tnnt3
Source: PLoS One. 2016 Aug 12;11(8):e0161096. doi: 10.1371/journal.pone.0161096 (PMC4982693; doi:10.1371/journal.pone.0161096)

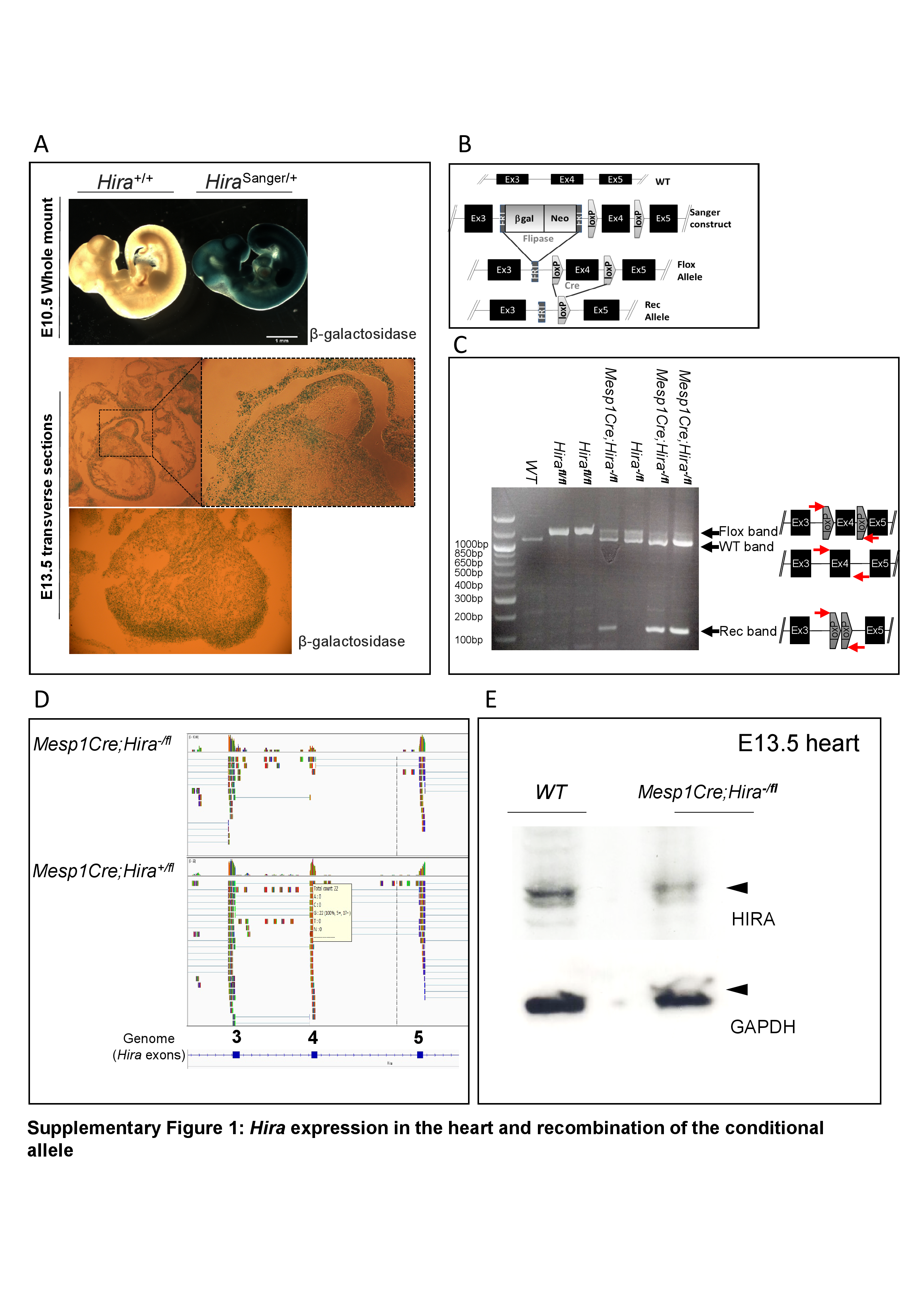

Supplement: S1 Fig — A. β-Galactosidase assay on E10.5 and E13.5 HiraSanger/+ and Hira+/+ embryos, either on whole mount or on heart sections as indicated, showing an ubiquitous expression of Hira. B. Schematic representation of the different Hira alleles used in the manuscript. C. Recombination of the floxed allele by Mesp1 driven CRE recombinase was assessed by PCR using DNA from the anterior limb which contains Mesp1 positive cells, detection of the various alleles is shown. D. Recombination was also demonstrated at the mRNA level by the reduced number of reads in RNAseq (22 in control and 3 in mutant). E. Reduced protein level of HIRA in Mesp1Cre;Hira-/fl hearts. This experiment was done by comparing pools of 3 Hira+/fl and 3 Mesp1Cre;Hira-/fl E13.5 hearts. Remaining protein is likely to originate from non-Mesp1-expressing cardiac linages (e.g. circulating cells and ingressing neural crest). (TIFF) [file pone.0161096.s001.tiff]

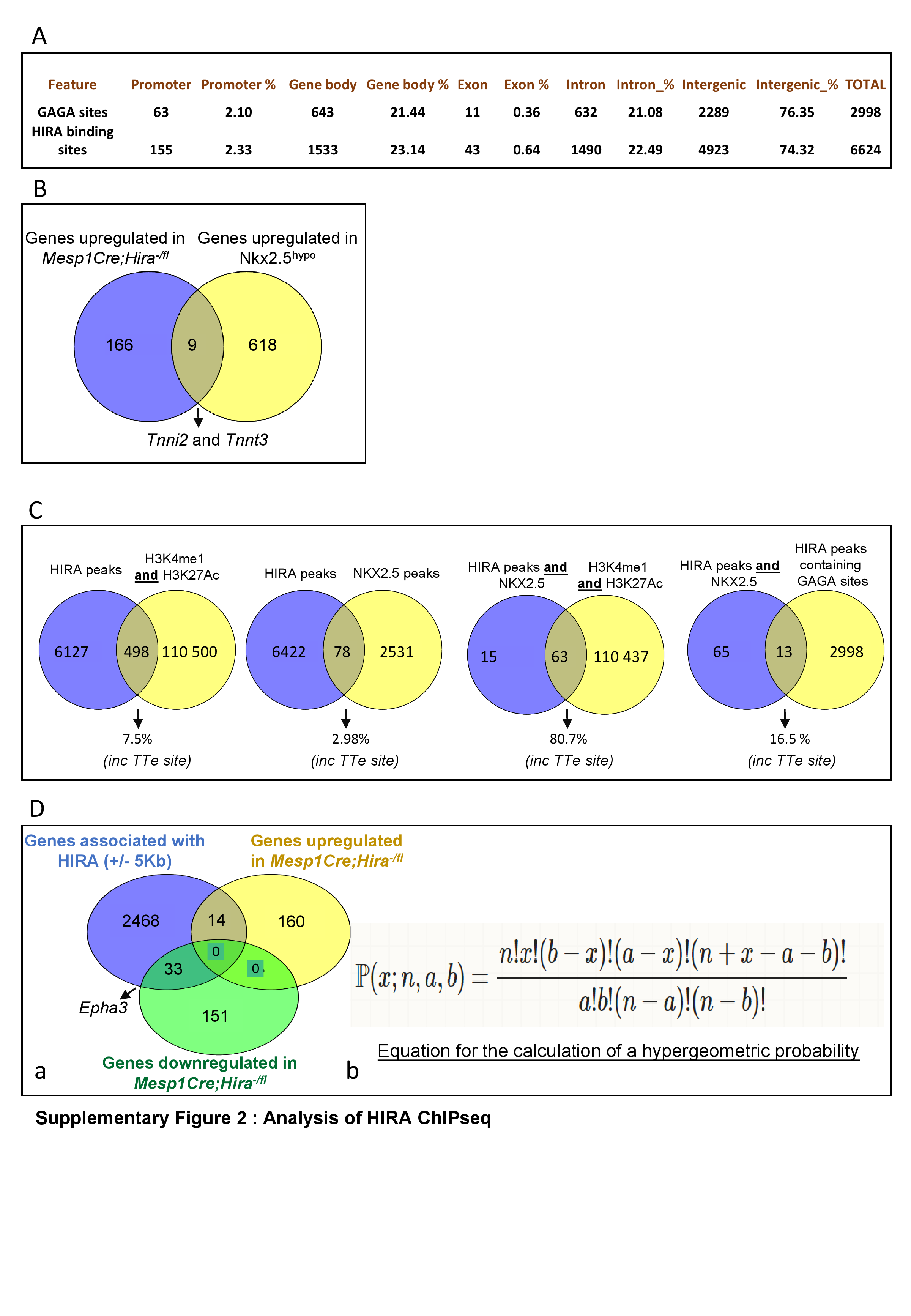

Supplement: S2 Fig — A. Table summarising the genic distribution of HIRA binding sites and the GAGA motifs across the genome. 45% of HIRA peaks were found to have a GAGA motif. B. Venn diagram displaying the number of genes upregulated in hearts of E12.5 Mesp1Cre;Hira-/fl embryos and in E11.5 Nkx2.5 hypomorph hearts [11]. C. Venn diagram displaying the number of overlaps between HIRA ChIPseq peaks at E12.5 (this study), enhancer signatures H3K4me1 and H3K27Ac at E13.5 (encode database ENCSR663VWL) and NKX2.5 ChIPseq peaks in E11.5 hearts [11] as indicated. D. a. Venn diagram displaying the overlap between genes whose expression is dysregulated in E12.5 Mesp1Cre;Hira-/fl hearts and genes which are enriched for HIRA in WT E12.5 hearts. The enrichment includes any peak in the gene body and/or within 5Kb upstream of the TSS or downstream of TES. b. The equation for the calculation of the probability of having 47 genes (x) in common between two independent groups: 2515 (b, HIRA ChIPseq genes) and 360 (a, RNAseq data) in the mouse genome which has approximately 22 000 genes (n). The result of this hypergeometric probability calculation is not significant: p(x > = 47) = 0.184. (TIFF) [file pone.0161096.s002.tiff]

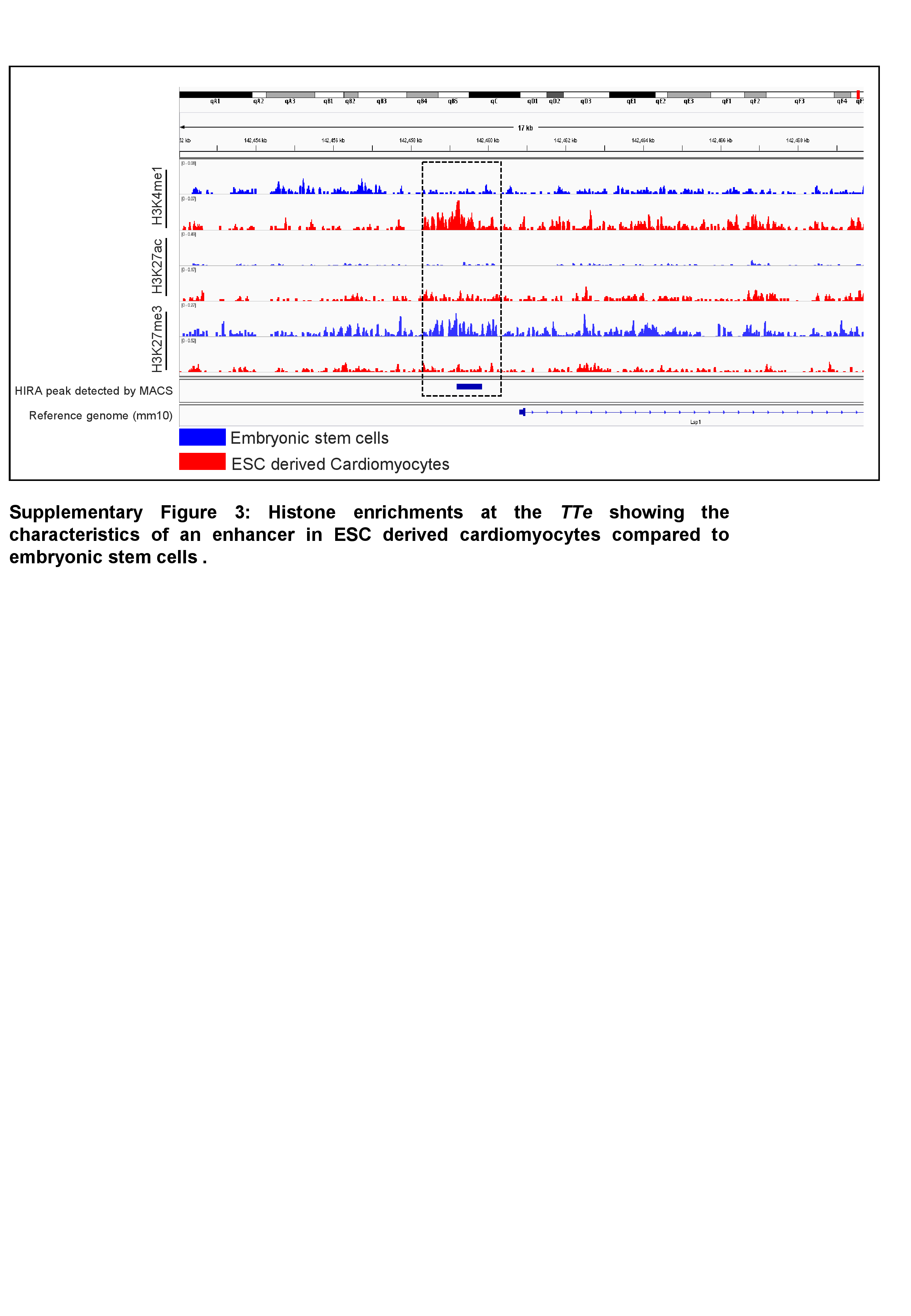

Supplement: S3 Fig — The enrichment of H3K4me1 and H3K27ac and the lack of repressive H3K27me3 modifications represent a distinct chromatin pattern observed in active enhancers (black box around the TTe). The development of this pattern mirrors the upregulation of Tnni2 seen during the differentiation process and supports the association of the TTe enhancer with its expression. Histone ChIpseq in ESC derived cardiomyocytes and ESCs were obtained from Wamstad and colleagues [24] (Gnomex accession numbers 44R and 7R2). (TIFF) [file pone.0161096.s003.tiff]

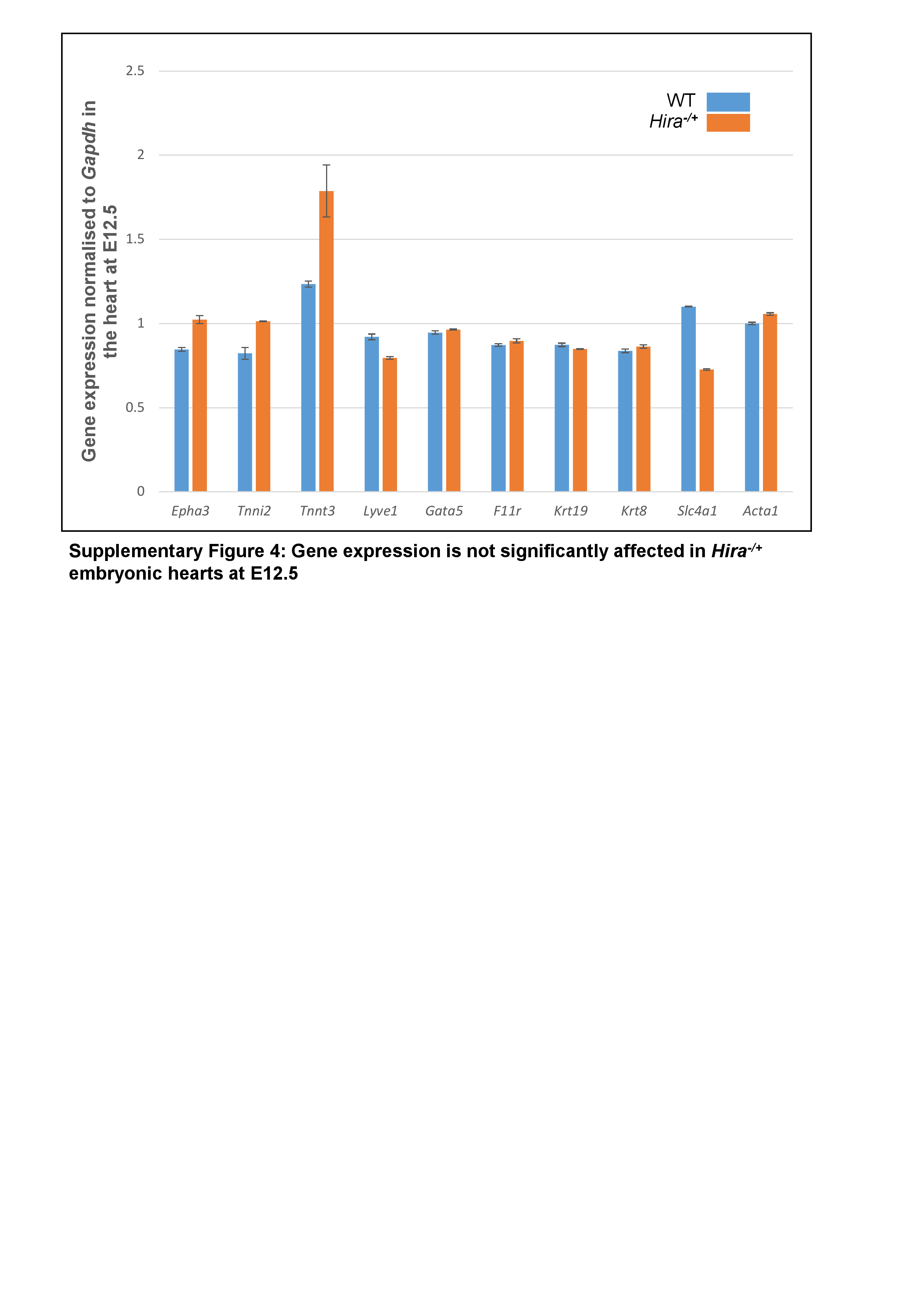

Supplement: S4 Fig — A subset of genes affected in Mesp1Cre;Hira-/fl hearts was quantified in Hira+/- mutant hearts using real time PCR. No significant changes were detected in Hira heterozygotes. (TIFF) [file pone.0161096.s004.tiff]

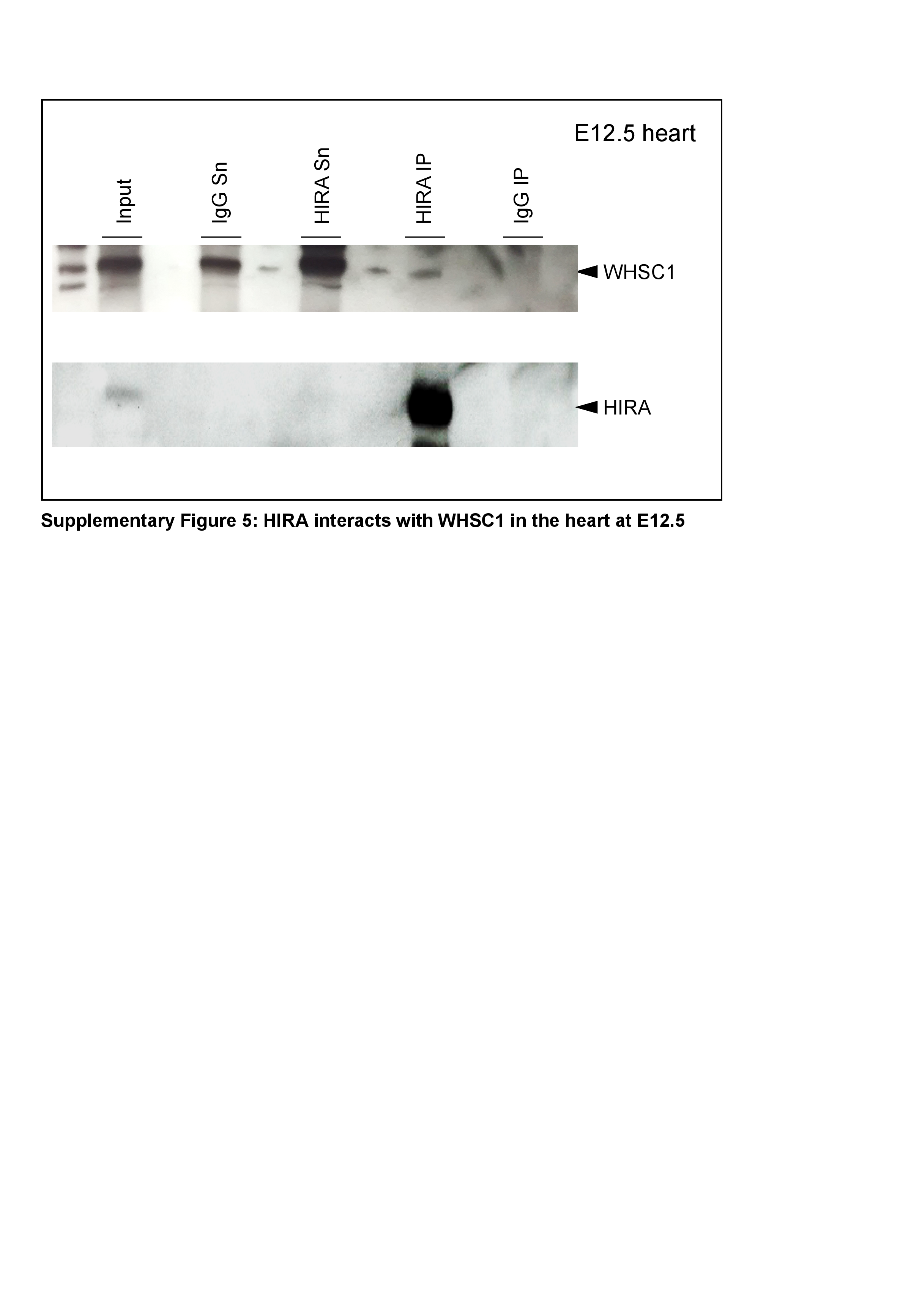

Supplement: S5 Fig — 30 embryonic hearts from WT embryos were isolated at E12.5, pooled and immunoprecipitated (IP) with anti-HIRA Wc15 antibody then immunoblotted with anti-WHSC1 antibody. Presence of HIRA in the IP was verified using anti-HIRA Wc119 antibody. (TIFF) [file pone.0161096.s005.tiff]

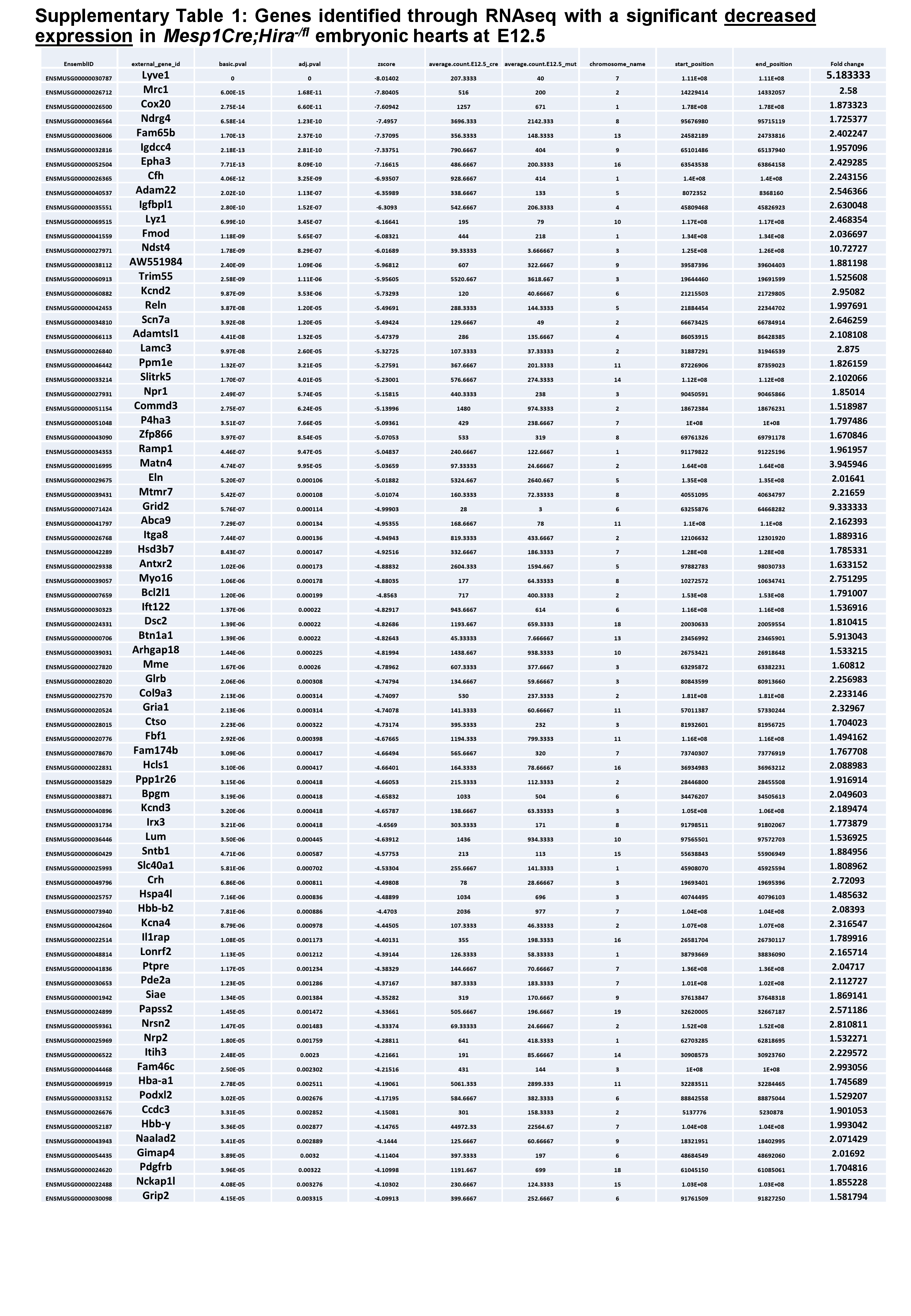

Supplement: S1 Table — (TIFF) [file pone.0161096.s006.tiff]

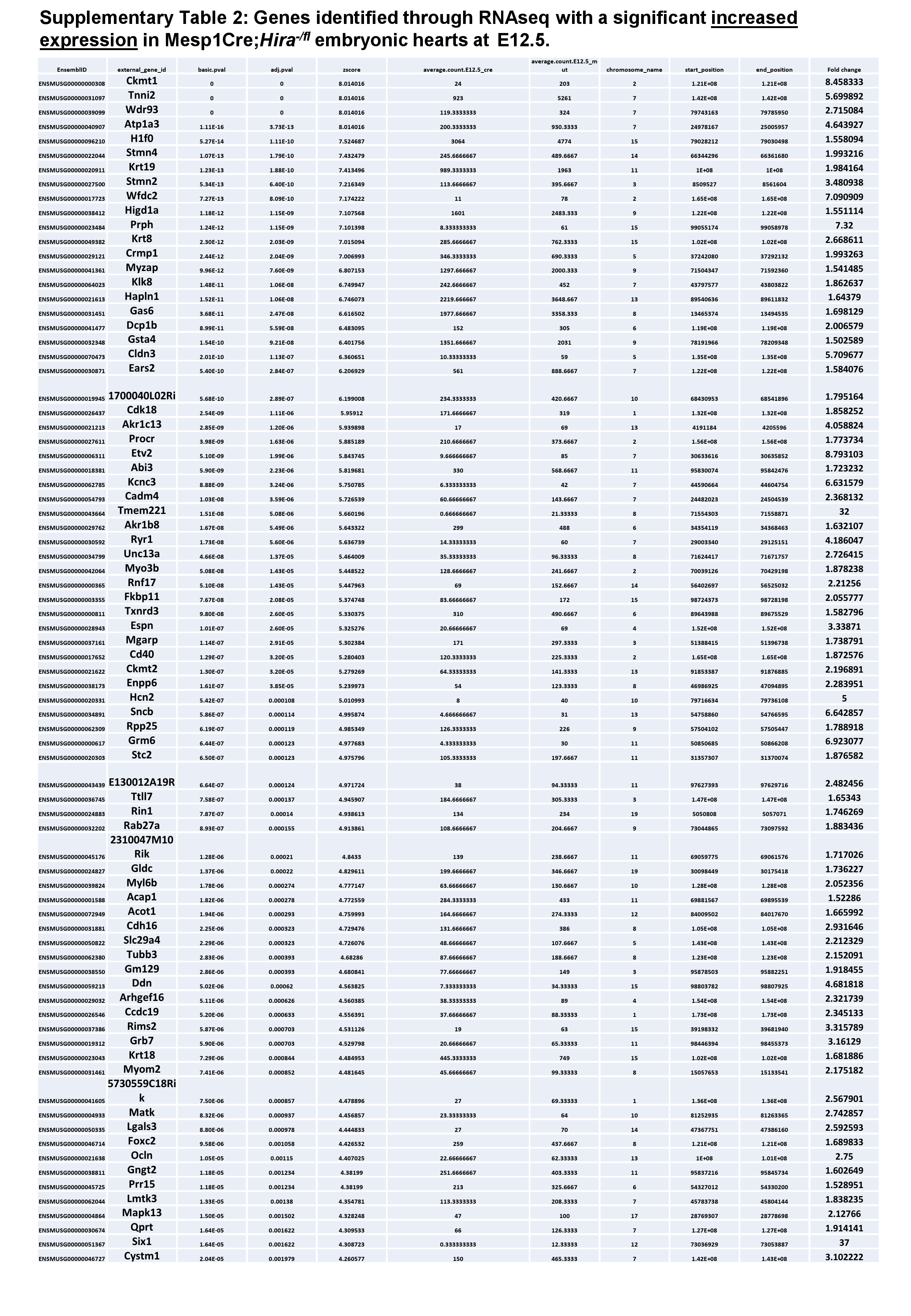

Supplement: S2 Table — Presented here are genes with the lowest p-value. The fold change is presented here as an absolute value. Test applied: Mann-Whitney unpaired, Benjamini Hochberg FDR, p ≤ 0.05, FC ≥ 1.5. The complete list can be accessed with at the GEO database under accession number GSE79937. (TIFF) [file pone.0161096.s007.tiff]

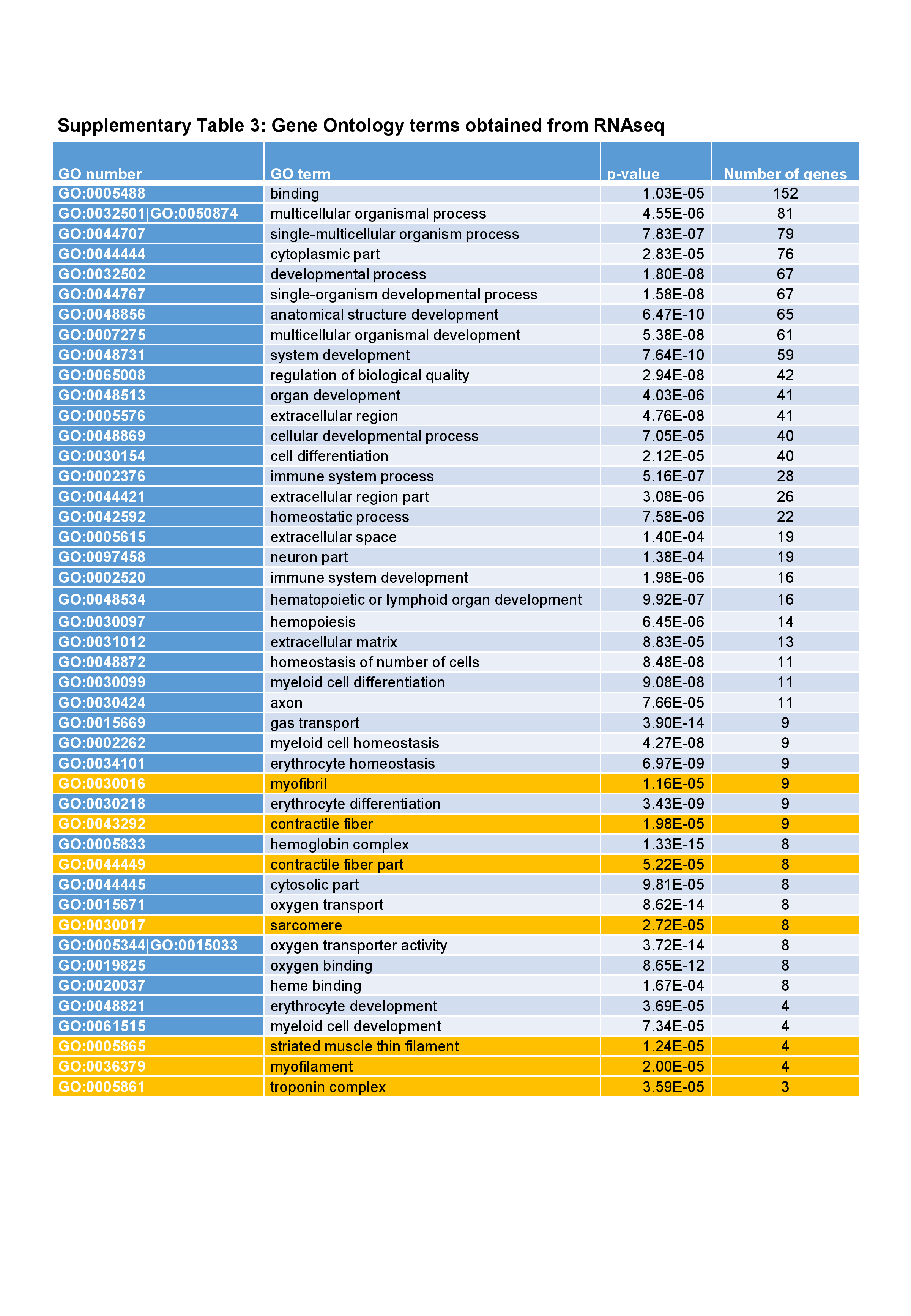

Supplement: S3 Table — GO analysis of the genes significantly dysregulated in Mesp1Cre;Hira-/fl embryonic hearts that have an over-representation of one or more GO terms that pass the cut-off p-value of 10−4. Terms relating to contractility and myofibril structure are highlighted in orange. (TIFF) [file pone.0161096.s008.tiff]

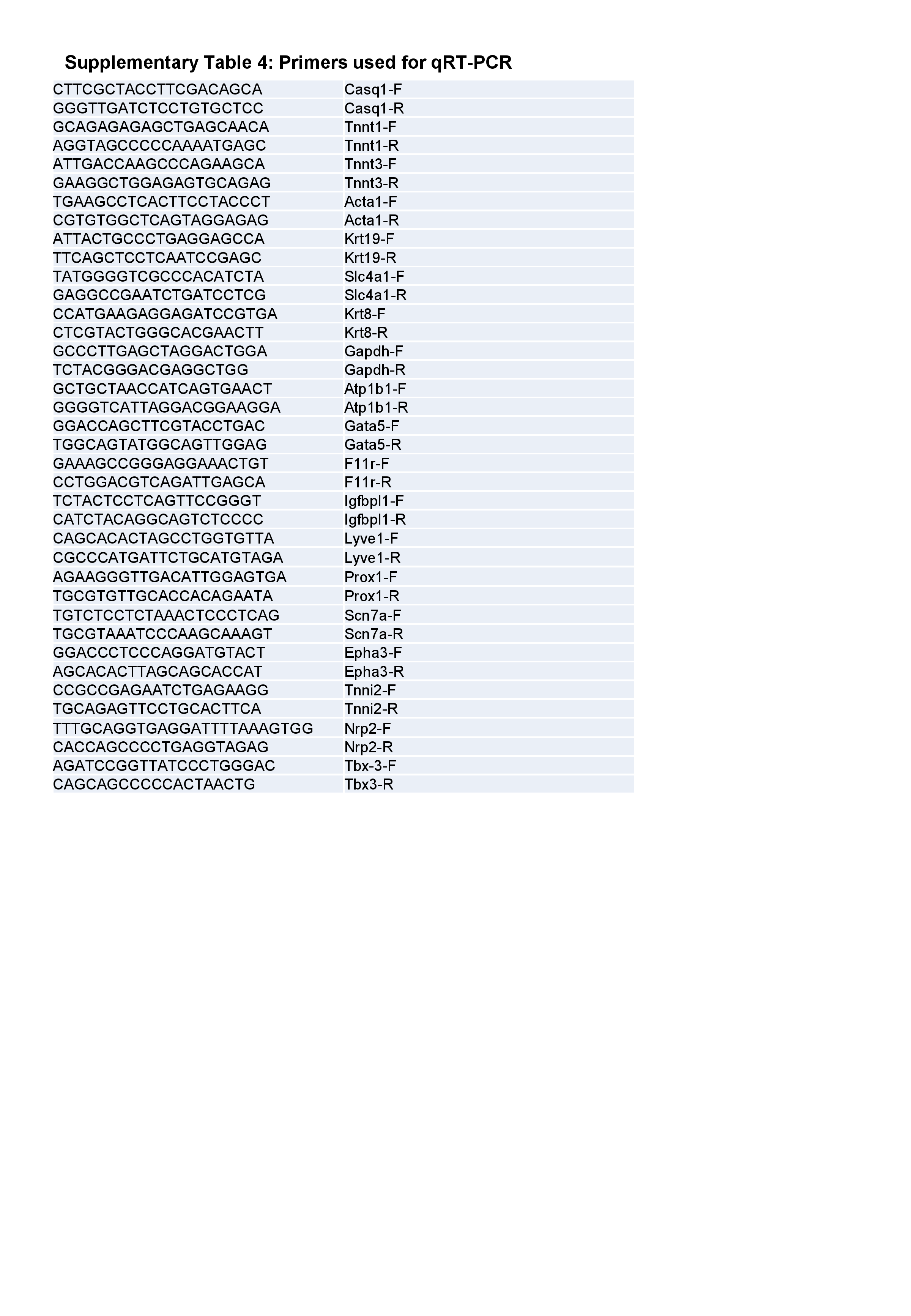

Supplement: S4 Table — (TIFF) [file pone.0161096.s009.tiff]

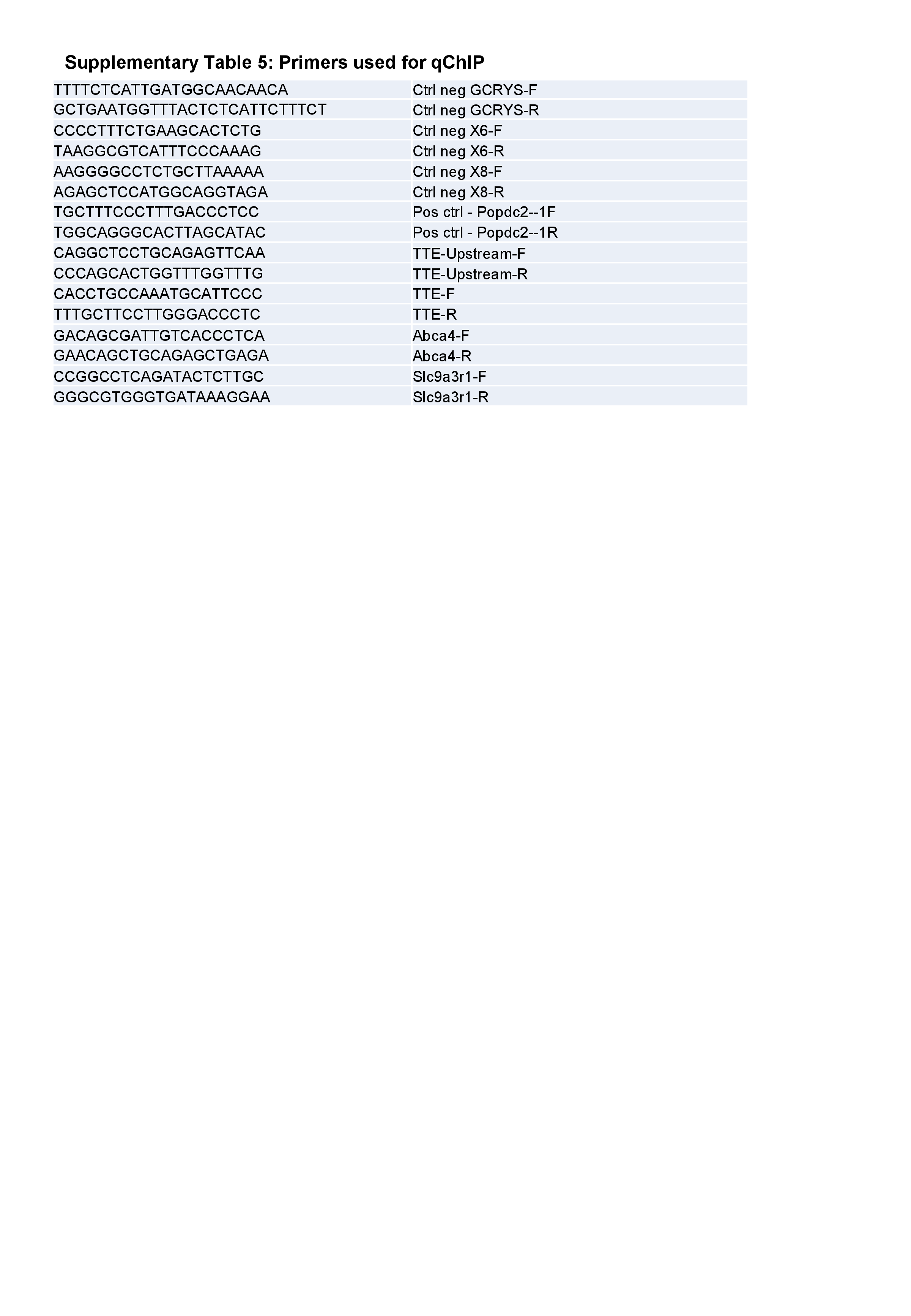

Supplement: S5 Table — (TIFF) [file pone.0161096.s010.tiff]
